# MS-based proteomic analysis of cardiac response to hypoxia in the goldfish (*Carassius auratus*)

**DOI:** 10.1038/s41598-019-55497-w

**Published:** 2019-12-12

**Authors:** Sandra Imbrogno, Donatella Aiello, Mariacristina Filice, Serena Leo, Rosa Mazza, Maria Carmela Cerra, Anna Napoli

**Affiliations:** 1 Dept of Biology, Ecology and Earth Sciences (BEST), University of Calabria, Arcavacata di Rende (CS), Italy; 2 Department of Chemistry and Chemical Technologies, University of Calabria, Arcavacata di Rende (CS), Italy

**Keywords:** Proteomics, Physiology

## Abstract

The exceptional hypoxia tolerance of the goldfish heart may be achieved through the activation of an alternative mechanism recruiting the first product of the anaerobic glycolysis (i.e. piruvate). This hypothesis led to design a classical mass spectrometry based proteomic study to identify in the goldfish cardiac proteins that may be associated with maintaining heart function under normoxia and hypoxia. A selective protein solubilization, SDS PAGE, trypsin digestion and MALDI MS/MS analysis allowed the identification of the 12 most stable hypoxia-regulated proteins. Among these proteins, five are enzymes catalyzing reversible steps of the glycolysis/gluconeogenesis network. Protein composition reveals the presence of fructose-1,6-bisphosphate aldolase B as a specific hypoxia-regulated protein. This work indicated that the key enzyme of reversible steps of the glycolysis/gluconeogenesis network is fructose-1,6-bisphosphate, aldolase B, suggesting a role of gluconeogenesis in the mechanisms involved in the goldfish heart response to hypoxia.

## Introduction

Molecular oxygen is essential for life. Hypoxia, a condition of insufficient oxygen supply to tissues, is common in natural environments, and is experienced by mammalian and non-mammalian vertebrates under both health and disease. In mammals, the hypoxia-dependent shift to anaerobic metabolism alters ATP production, thus reducing the animal’s ability to meet cellular requirements. This energy unbalance leads to death within few minutes.

Despite the critical importance of aerobic respiration to the maintenance of metabolic function, several animal species have solved the problem of hypoxia, thus being able to inhabit hypoxic, and even anoxic, environments^1,2^. This is the case of various fish, frog, and turtle species that tolerate anoxia, and of some snakes and insects that can endure severe hypoxia^3^. A variety of strategy to enhance O_2_ uptake have been reported. These include increases of respiratory surface area^4^, Hb synthesis and concentration in the blood^5,6^, haematocrit^7,8^, Hb–O_2_ affinity^7^, ventilation frequency and amplitude^9–12^, as well as redistribution of blood supply to critical tissues^13^. Moreover, a general rapid mechanism to enhance hypoxic survival is represented by metabolic rate depression (MRD)^14,15^. MRD, by occurring at both whole-animal (e.g. locomotion, reproduction, feeding) and cellular (e.g. growth, repair, protein synthesis) level^16,17^, quickly reduces ATP demand, rates of anaerobic fuel depletion (glycogen) and waste accumulation (lactate and protons).

Many aquatic systems undergo to environmental hypoxia. It is becoming progressively prevalent, severe and enduring because of anthropogenic and climate change effects. Among fish, *Carassius auratus* and *Carassius carassius* have an extraordinary ability to survive and remain active for long periods under hypoxia, even tolerating anoxia^1^. Thus, they have been recently regarded as suitable models to study physiological strategies that allow survival when O_2_ availability becomes a limiting factor^18^. Some of the adaptive mechanisms evolved by these fish species include the capacity to produce ethanol as an alternative, acid-base neutral, end product^19^, and to maintain heart activity using anaerobic metabolism as energy source^20^. Moreover, under acute hypoxia, the goldfish is characterized by an improved cardiac performance and sensitivity to heterometric regulation^21^ (i.e. the ability to enhance contractility in response to increased preload), which may favour functional and metabolic interactions between organs and tissues involved in hypoxia tolerance^22^. Of note, it has been recently reported that, differently by other hypoxia resistant species, goldfish can maintain routine MR at severely hypoxic PwO_2_ values, MRD representing a key mechanism of anoxic, but not of hypoxic, survival in goldfish^23^. In this species, hypoxia exposure results in a transient increase in lactate, which subsequently (within few hours) recovers to values similar to the normoxic controls^23^.

Based on these premises, our idea is that the exceptional hypoxia tolerance of the goldfish heart may be achieved through the activation of mechanisms which allow a reutilization of the first product of the anaerobic glycolysis (i.e. pyruvate). To test our hypothesis, we examined the cardiac proteomes, and evaluated the pyruvate concentration and the lactate dehydrogenase enzymatic activity in both normoxic and hypoxic goldfish hearts. A MS-based proteomic study was planned to identify cardiac proteins that might be associated with maintaining heart function at normoxic and hypoxic conditions in the goldfish. A simple procedure for selective protein solubilization, SDS PAGE, trypsin digestion and MALDI MS/MS analysis was applied to normoxic and hypoxic goldfish cardiac tissues.

## Results

**Figure 1:**
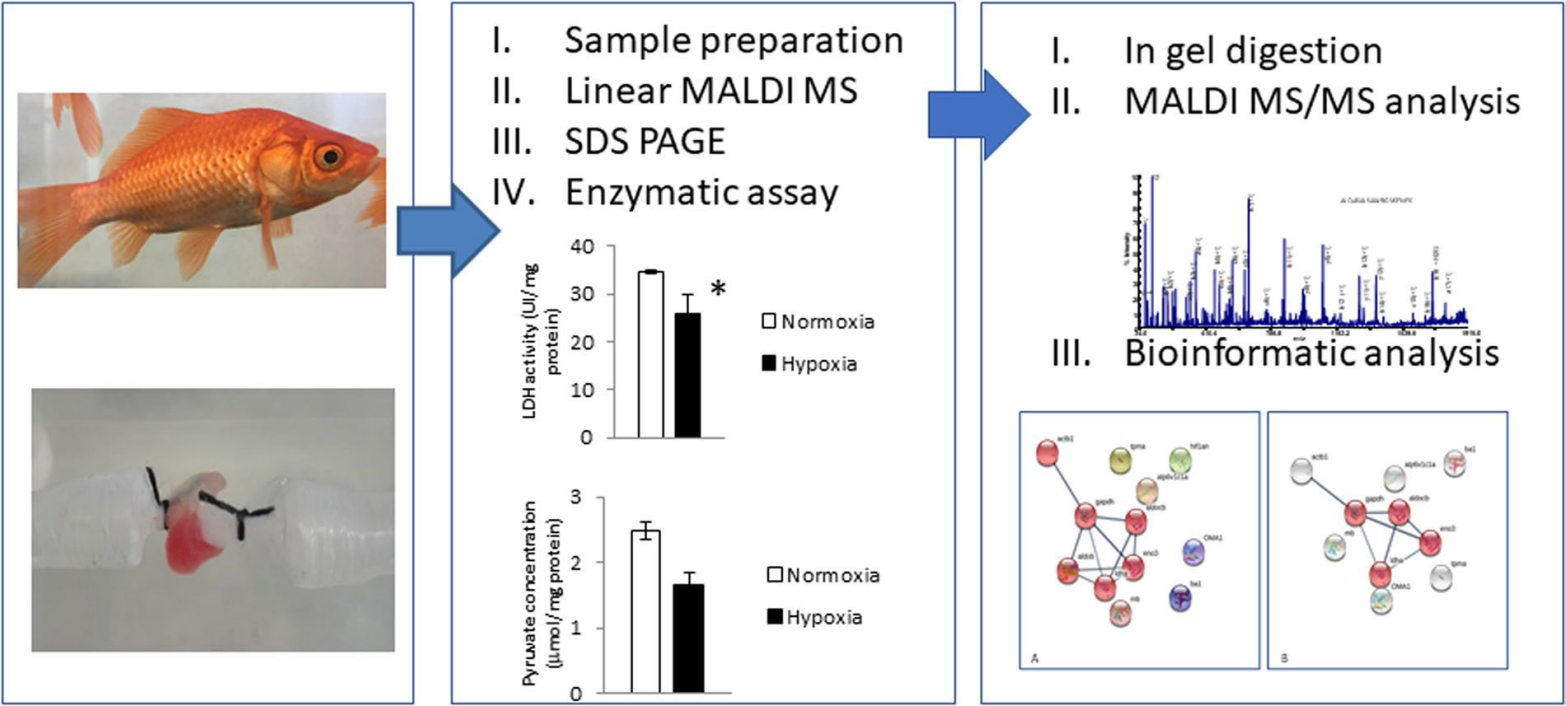
Experimental strategy.

To explore the possible functional roles (e.g., enzyme or biomarker) of the candidate proteins involved in goldfish cardiac adaptation to hypoxia, we established the experimental strategy outlined in Fig. 1. The strategy has two steps: MALDI MS, SDS MALDI MS/MS analysis and enzymatic assay on cardiac homogenates from goldfish hearts perfused under normoxic and hypoxic conditions. Enzymatic assay, i.e. pyruvate and lactate dehydrogenase assay, were used as orthogonal classic approach to validate mass spectrometry and bioinformatics output on cardiac sub proteome.

### Protein extraction and identification

Few proteomic studies have been stated on goldfish^24^ using two-dimensional (2D) gel electrophoresis followed by in-gel digestion and mass spectrometric analysis^25,26^. Literature data revealed the tri-modality feature for zebrafish (*Danio rerio*) heart proteome, with the majority of proteins centered around pIs 5.5 and 9 and fewer proteins around neutral pI^27^. Furthermore, proteomic data on *Gillichthys mirabilis* cardiac tissues revealed 37 protein, involved in energy metabolism, mitochondrial regulation, iron homeostasis, cytoprotection against hypoxia, and cytoskeletal organization, with the majority of proteins centered around pIs 5.5–6.5^28^ The reliable detection of species-specific proteins and peptides, unique in mass and amino acid sequence, depends on proper protein solubilization, digestion, and sensitive MS analysis^29,30^. Therefore, two different aqueous media were tested for the ability to extract proteins from the goldfish cardiac tissues: phosphate buffer (pH 7.5) and ammonium bicarbonate solution (50 mM, pH 8). Proteins from goldfish cardiac tissues extracted with the two media were compared on 1-DE (Fig. 1S). A simple band pattern was obtained from both extracts with a low number of protein bands in each extract. The observed selective protein patterns generated by each extraction solutions emphasize the necessity of careful selection of the medium. Band patterns did not reveal significant differences among the extraction, suggesting a tissue specific sub-proteome denoted by chemically homogeneous protein families^31–33^.

The rapid and significant upgrading of sensitivity, throughput and mass accuracy of modern mass spectrometers drastically improved gel-free proteomic approaches. It is known that the accuracy of gel electrophoresis is frequently too low to be useful for mass measurement of intact proteins^34^. MALDI MS has successfully been used for the direct analysis of peptides and proteins in biological tissues^35–38^, and the MS profiling has been applied to identify condition specific protein patterns in fish tissues and organs^39^. The molecular distribution acquired by MS provide important data to highlight physiological responses associated to environmental changes^39^, and the MS-based chemical component profiling represents a powerful tool to obtain precious information. The direct MS analysis of tissue extracts has been found to be a useful and robust approach to identify features^40^ (m/z value and their relative peak heights/areas) within spectra that differentiate between distinct groups (e.g., spectra derived from normoxic vs hypoxic conditions). Moreover, the direct desorption of proteins from crude extracts shows the advantage of minimizing sample loss during its workup. The selection of an appropriate matrix/sample preparation method is necessary to obtain high-quality mass spectra from nanomolar (50–100 nM) protein samples. The singly protonated molecule [MH]^+^ is predominant ion species in the MALDI mass spectrum, however multiply charged ions (doubly and triply charged [M + 2H]^2+^, [M + 3H]^3+^) are progressively evident as protein molecular mass increases. It is known that matrices with higher ionization energy, such as α-cyano-4-hydroxycinnamic acid (CHCA), generate multiply charged protein ions. For the direct MS analysis of crude extracts presented here, CHCA (5 mg/mL; sample/matrix: 1/2 (v/v)) was chosen in order to obtain the highest analyte sensitivity, favoring the formation of doubly and triply charged protein ions. The formation of multiply charged ions in linear MALDI experiments represents a valuable advantage for the accurate protein mass determination. Although chemical composition and molecular mass affect the ionization efficiency, and consequently the relative peak intensities, the magnitude of the MS signals reflects the relative amounts of mixture components. The relative low number of protein ion signals which can be detected in spectra agrees the experimental protocol adopted for tissue extraction.

**Figure 2:**
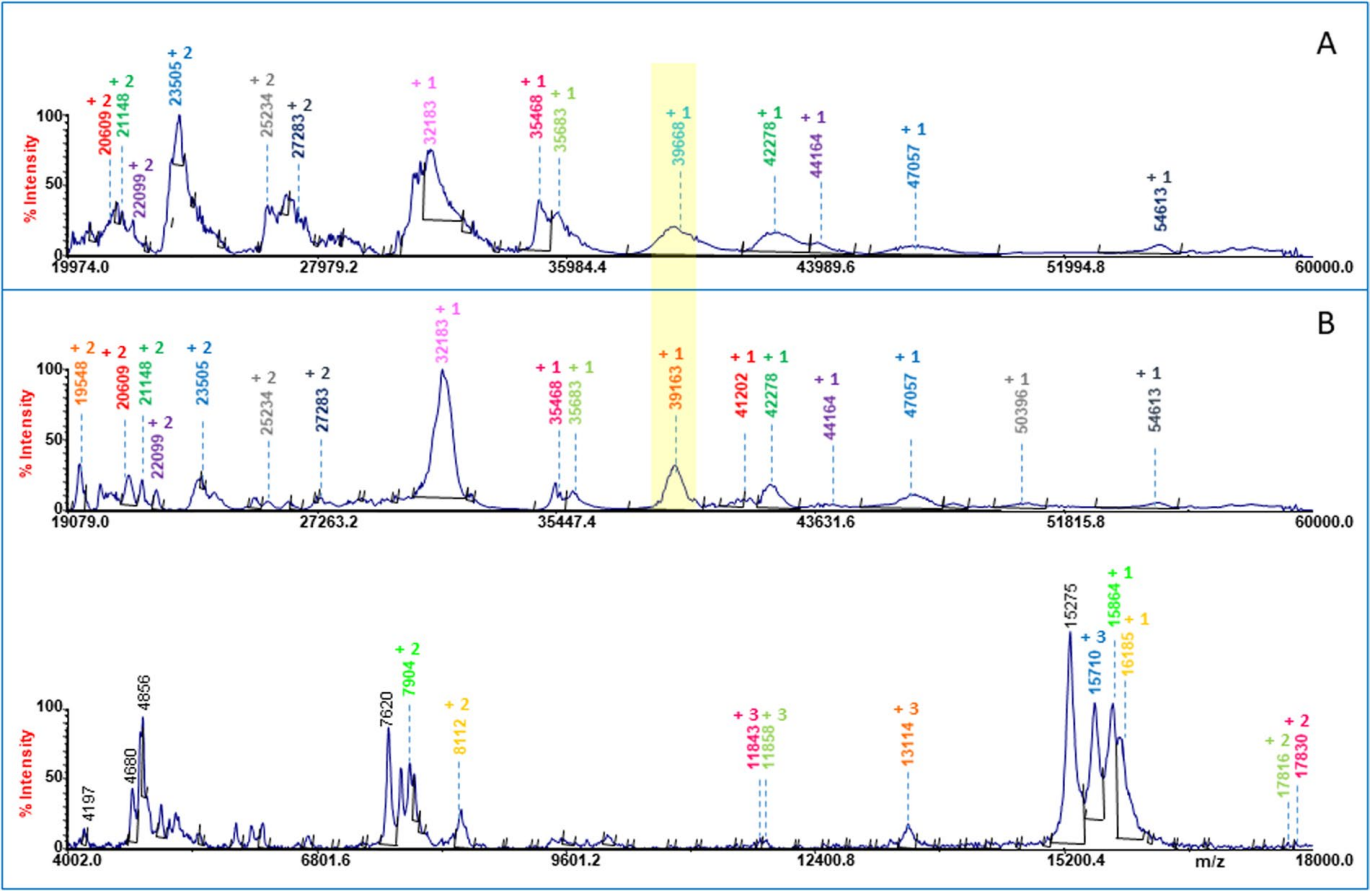
Linear MALDI MS of normoxic (**A**) and hypoxic (**B**) cardiac tissue extract.

Representative linear MALDI MS spectra are reported in Fig. 2A,B. Figure 2B displays linear MALDI mass spectrum of hypoxic cardiac tissue extract (4 ÷ 60 kDa). The presence of single and multicharged protein ions allowed the identification of twelve proteins with 0.1% ÷ 0.6% accuracy (Table 1, column 1). In order to compare protein profiles of hypoxic and normoxic cardiac tissue extracts, the obtained MALDI-MS spectra were exported as ASCII spectra (S/N = 5; Data Explore 4.1). The spectra were aligned by setting centroid peak shift to 0.1% of the mass over charge (m/z) value, and the average molecular mass (using +1, +2 and +3 ion signals) of each mixture component was determined. The protein hypoxic and normoxic profiles exhibited such a few differences, the most relevant was found in the mass range between 38.5 kDa and 40 kDa. The protein signal of m/z 39163 ± 153 Da detected in hypoxic cardiac tissue extracts was absent in normoxic tissue extracts, which instead exhibited a protein signal at 39668 ± 83 Da. The difficult matching of the experimentally determined and theoretical molecular weights of hypothetical proteins, as predicted through genome annotations, could represent a limitation. Several factors may cause this discrepancy, and they include polymorphism in the protein sequence between the species, incorrect genome annotation, redundancy or errors in the amino acid sequences, and possible post-translational modifications. The top-down approach utilizing MS/MS experiments on single and double charged ions signals did not allowed the identification of proteins. The concentration of proteins present in low amounts in the extracts, limits the applicability of top-down experiments even though the mixtures obtained from tissue extractions showed a low complexity. Despite the above technical restrictions in the full identification of the cardiac proteins (which was beyond the scope of this work), the results demonstrate that MALDI-MS can suitably be used in the direct analysis of tissue extracts, allowing the detection of the specific protein pattern associated to the physiological conditions of each individual.

**Table 1:** Protein identified by MALDI MS experiments and after in gel digestion- MALDI MS and MS/MS.

	MW MS	MW^a^	PI^b^	Protein ID	*Name*	Normoxia	Hypoxia
1	44164 ± 45	44315	6.93	VTC1A_DANRE	*V-type proton ATPase subunit C 1-A*	+	+
2	47057 ± 62	47287	6.61	ENOB_SALSA	*Beta-enolase*	+	+
3	39163 ± 153	39288	8.48	ALDOB_ DANRE	*Fructose-bisphosphate aldolase B*	−	+
4	39668 ± 83	39449	6.41	ALDO_CARAU	*Fructose-bisphosphate aldolase C*	+	−
5	42278 ± 22	41971	5.23	ACTS_CARAU	*Actin, alpha skeletal muscle*	+	+
6	32183 ± 49	32723	4.70	TPM1_DANRE	*Tropomyosin alpha-1 chain*	+	+
7	35468 ± 224	35784	8.19	G3P_DANRE	*Glyceraldehyde-3-phosphate dehydrogenase*	+	+
8	35683 ± 147	36334	6.70	LDHA_FUNHE	*L-lactate dehydrogenase B-A chain*	+	+
9	15864 ± 82	15851	7.89	B3CJI6_CARAU	*Myoglobin*	+	+
10	16185 ± 52	16210	7.85	HBB_CARAU	*Hemoglobin subunit beta*	+	+
11	54613 ± 69	54050	9.69	OMA1_DANRE	*Metalloendopeptidase OMA1, mitochondrial*	+	+
12	41202 ± 21	39992	5.66	HIF1N_DANRE	Hypoxia-inducible factor 1-alpha inhibitor	−	+
13	50396 ± 140	—	—	Unknown		−	+

^a,b^https://www.uniprot.org/.

Protein gel image analysis revealed 7 distinct protein bands in both groups (i.e. normoxic and hypoxic goldfish cardiac tissues, Fig. 1S). Tryptic peptides obtained by in gel digestion were loaded on to MALDI plate and analyzed in MS and MS/MS mode. The identification of protein parent was performed using Protein Pilot Paragon Method and the results of the database search are listed in Table 1. MS/MS data were processed using a mass tolerance of 0.2 Da and 10 ppm for the fragment ions and precursor, respectively. The phosphorylation of serine and threonine was included in the variable modifications and all spectra were manually evaluated. From the two set of protein bands, we were able to identify 12 proteins using mass spectrometry (Table 1).

The complete lists of peptides and proteins for each of two different group of cardiac tissue are available in the supplemental data Tables 1S–12S. The detection of well-known hypoxia-induced proteins (up-regulated) such as glyceraldehyde-3-phosphate dehydrogenase (GAPDH), L-lactate dehydrogenase (LDHA), phosphoglycerate Hypoxia-inducible factor 1-alpha inhibitor (HIF1N), Tropomyosin alpha-1 chain (TPM1) and beta-enolase (ENOB) was indicative of the robustness of our experimental approach.

### Bioinformatic analysis

Gene ontologies and pathways analysis by PANTHER revealed the presence of 4 different protein classes. The most prominent classes were cytoskeleton proteins (25%), hydrolases (25%), lyases (12,5%), oxidoreductases (25%) and transporters (12,5%). The identified proteins were listed using the gene name and the ZFIN ID for the analogous reference species *Danio rerio* (Tables 13S and 14S). The final dataset was subsequently investigated by gene ontology, network analyses. The metabolic pathways were investigated by DAVID tools. The top score pathways included proteins involved in (a) glycolysis/gluconeogenesis, (b) biosynthesis of antibiotics, (c) biosynthesis of amino acids, (d) carbon metabolism, and (e) metabolic pathways. A large group of proteins (45%) was found to be involved in glycolysis/gluconeogenesis pathway. Proteins associated with this pathway were *aldocb, eno3, gapdh, ldha*. The study of functional domains by InterPro Motifs performed by the DAVID software discovered that the top protein motifs corresponded to globin and globin-like structural domains. Globins are heme-containing proteins ubiquitously expressed in vertebrates, including fish, in which they play a broad range of biological functions, directly or indirectly related to the tight control of oxygen levels and its toxic products^41^. The biological processes associated with the identified proteins of goldfish were (a) glycolytic process, (b) response to hypoxia, (c) glucose metabolic process (Table 15S). Evaluation of the biological functions by STRING revealed 8 top biological processes (Table 15S), including (a) carbohydrate metabolic process, (b) response to hypoxia, (c) pyruvate metabolic process, (d) glycolytic process. The comparison between normoxic and hypoxic goldfish cardiac proteome evidenced the “carbohydrate metabolic process” and “response to hypoxia” as the major biological processes. These results are in accordance with the high number of enzymes (5) identified.

**Figure 3:**
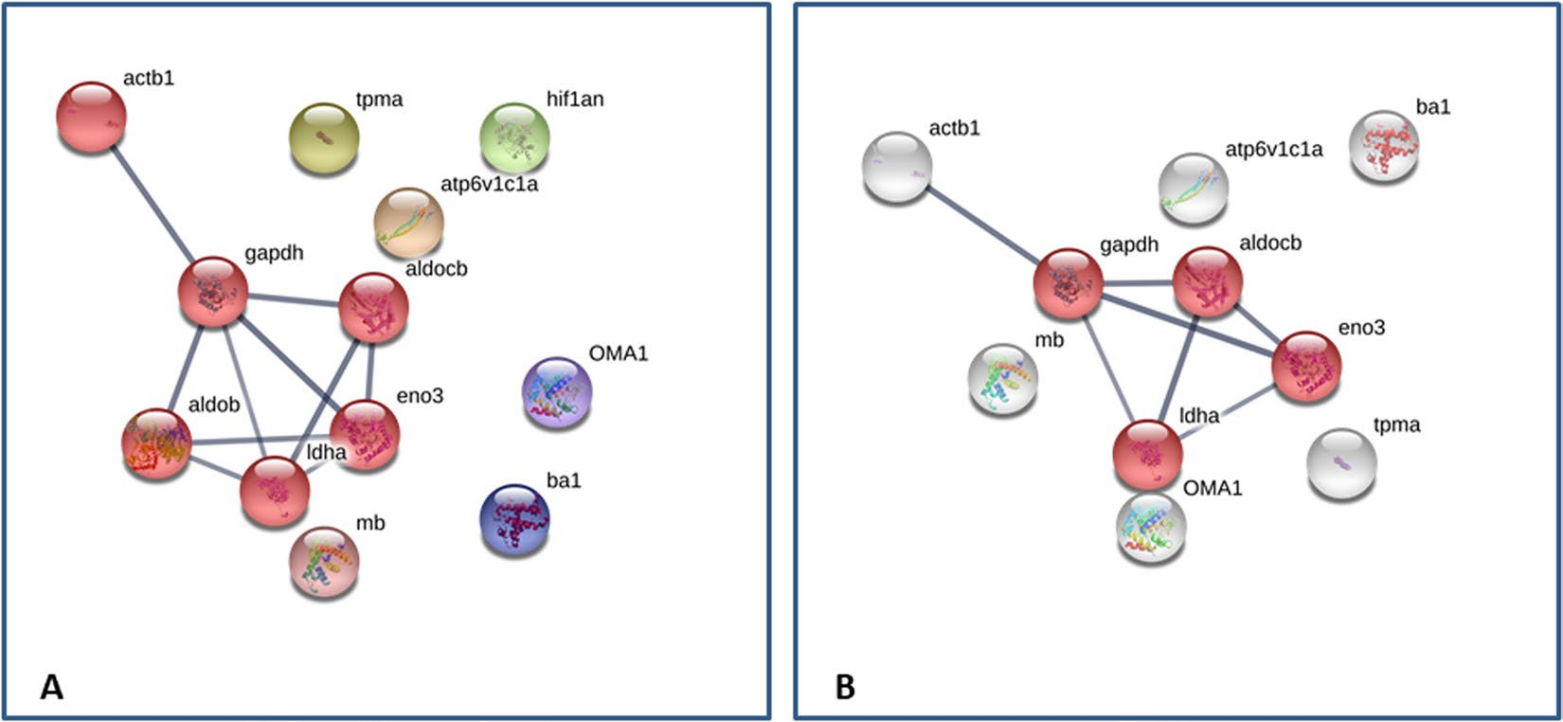
Protein-Protein interaction network by STRING software (panel A: Hypoxia; panel B: Normoxia).

All proteins identified for both hypoxic and normoxic proteome were subjected to STRING database (https://string-db.org/). Only the interactions tagged as “high confidence” (0.7) in STRING database, have been considered to minimize false positives as well as false negatives. For hypoxia, the network is composed by 12 nodes (proteins) and 10 edges (interactions); expected number of edges 0 and PPI enrichment p-value: 9.52e-13 (Fig. 3). To create this network, a value of 3 for the MCL clustering coefficient was chosen.

### Enzymatic assay to validate mass spectrometry and bioinformatic results on cardiac sub proteome

**Figure 4:**
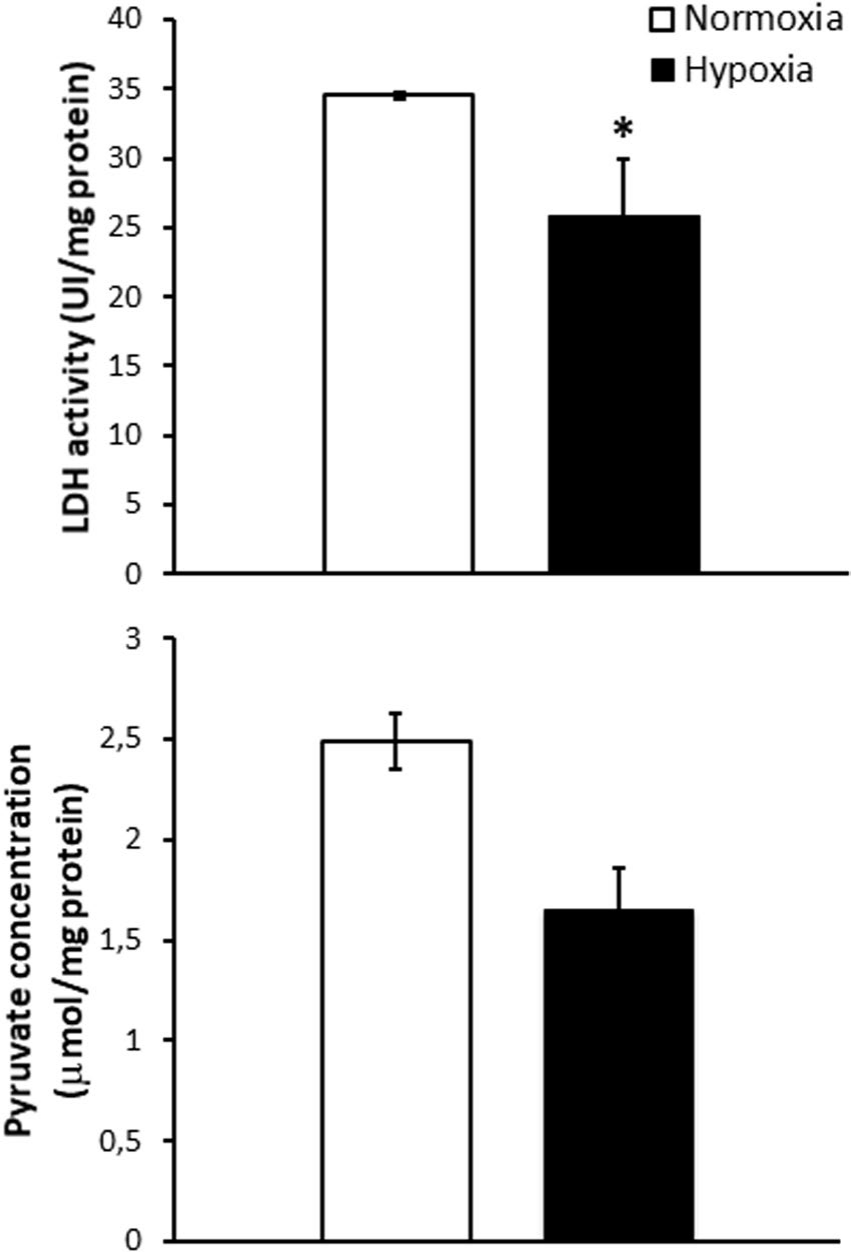
LDH activity (upper panel) and Pyruvate concentration (lower panel) in goldfish (*C. auratus*) hearts perfused either under normoxic or hypoxic conditions. Values are the mean ± S.E.M. of three individual experiments for each condition. Statistics are assessed by *unpaired t-test* (*p < 0.05).

To validate mass spectrometry and bioinformatic results on cardiac sub proteome, we measured pyruvate concentration and LDH activity in homogenates of goldfish hearts perfused either under normoxic or hypoxic conditions. Results showed that, with respect to the normoxic counterpart, lower levels of LDH activity were measured in homogenates of hearts exposed to hypoxia (hypoxia: 25.9 ± 4 UI/mg protein; normoxia: 34.56 ± 0.24 UI/mg protein; Fig. 4). This hypoxia-dependent reduction of LDH activity resulted associated to a slight, although not significant, reduction in pyruvate levels (hypoxia: 1.65 ± 0.21 μmol/mg protein; normoxia: 2.49 ± 0.84 μmol/mg protein; Fig. 4). This suggests that under hypoxia, the conversion of pyruvate to lactate is less effective; however, it is not associated with pyruvate accumulation.

## Discussion

In the study here reported, mass spectrometry followed by bioinformatics analysis and enzymatic assays has been used to study *ex vivo* proteome of normoxic and hypoxic goldfish cardiac tissues. MS-based proteomics is a rapid and sensitive tool for the identification^42^ and quantitation^43–45^ of proteins in specific functional contexts. The experimental approach here reported was designed with the purpose to identify a set of the most stable known hypoxia-regulated proteins. This functionally oriented strategy, focused on the search for acid hydro-soluble proteins, was driven by the hypothesis that the exceptional hypoxia tolerance of the goldfish heart may be accomplished through the activation of alternative mechanisms. MS/MS analysis of goldfish hearts sub-proteome has allowed to identify the hypoxia-regulated protein composition under both normoxia and hypoxia (Table 1).

**Figure 5:**
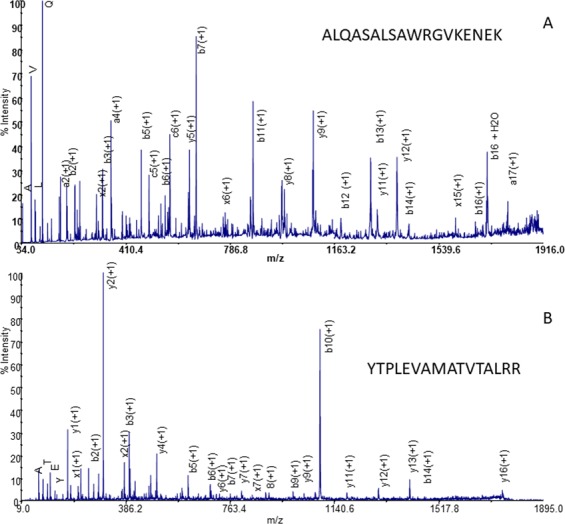
MALDI MS/MS spectrum of (**A**) m/z 1958.06 ([M − H] ^+^) and (**B**) of m/z 1791.99 ([M − H]^+^).

Among the well-known hypoxia-induced proteins, we identified a novel hypoxia-regulated protein fructose-bisphosphate aldolase B (ALDOB_ DANRE), which was found to be expressed in *ex vivo* hypoxic cardiac samples. In particular, MS/MS assignment of 40 kDa protein spots from hypoxic and normoxic conditions corresponds to fructose-bisphosphate aldolase B (ALDOB_ DANRE) and fructose-bisphosphate aldolase C (ALDOC_ CARAU), respectively. Sequence information was obtained with tandem MS of 18 peptides (Table 6S). These peptides fully match the aldoc (ALDOC_ CARAU) sequence and cover 51% of the 363-amino acid protein (Table 6S). Similarly, 18 peptides match the aldob (ALDOB_ DANRE) sequence and cover 68% of the 364-amino acid protein (Table 5S). Aldob and aldoc are isoforms of fructose-bisphosphate aldolase sharing 73.901% sequence identity. The relatively large number of amino acid differences between aldoc and aldob (e.g. aldoc has 95 mutations compared with the aldob enzyme) make straightforward the identification of the two enzymes. For example, the point mutations of ^315^K → R, ^317^Q → V, ^318^A → K, ^319^A → E and ^322^K → E (aldob *vs* aldoc) were univocally assigned by a single MS/MS experiment. The fragmentation pattern of the sequence ^305^ALQASALSAWRGVKENEK^322^ ([M − H]^+^ of m/z 1958.06, Δppm = 5.9; Fig. 5A) shows the complete b-type ion series allowing the identification of ALDOC_CARAU in normoxic condition. Similarly, the detection of the ion of m/z 1791.99 (Δppm = 5.7; Fig. 5B) from 40 kDa digested obtained in hypoxic condition allowed the identification of aldob. The sequence ^244^YTPLEVAMATVTALRR^259^, deduced from the fragmentation pattern of m/z 1791.99, featured the point mutations at the positions 244 (F → Y), 245 (S → T), 246 (N → P), 247 (L → Q) and 248 (I → V) (aldoc *vs* aldob). Therefore, the aldob (ALDOB_DANRE) was assigned as the protein source of the peptide sequence ^244^YTPLEVAMATVTALRR^259^ on the basis of the sequence congruency with the SwissProt database.

In mammalian tissues, three aldolase isoforms have been identified: aldolase A, the classical muscle enzyme; aldolase B, a form predominantly expressed in the liver; aldolase C, whose expression has been observed in the brain. Unlike aldolase A, which appears to be more effective in participating to glycolysis and aldolase C which, although possessing intermediate properties, seems to be utilized mainly in the cleavage of fructose-1,6-bisphosphate, aldolase B has evolved to have a role in gluconeogenesis^46–49^. Metabolic state and substrates availability have been proposed to modulate the expression of aldolase isoforms, and thus of glucose synthesis or degradation^50^. The protein network highlighted in Fig. 3A showed the main set composed by 5 nodes (*aldob, aldocb, eno3, gapdh, ldha*) which results in accordance with the top canonical pathway previously obtained by DAVID. These nodes are referred to enzymes catalyzing reversible steps of the glycolysis/gluconeogenesis network. No interaction was observed for the hypoxia-inducible factor 1-alpha (HIF-1α) inhibitor (FIH-1). HIF-1α is the major transcriptional regulator of the cellular hypoxia response. Its binding to DNA as an α/β heterodimer is regulated by oxygen dependent prolyl (PHD) and asparaginyl (FIH-1) hydroxylases. PHD is involved in the stability of HIF–α sub-unit, while FIH-1 regulates its transcriptional activity. Unlike PHD enzyme, whose activity is completely suppressed by hypoxia, FIH-1 remains active even at relatively low oxygen levels.

**Figure 6:**
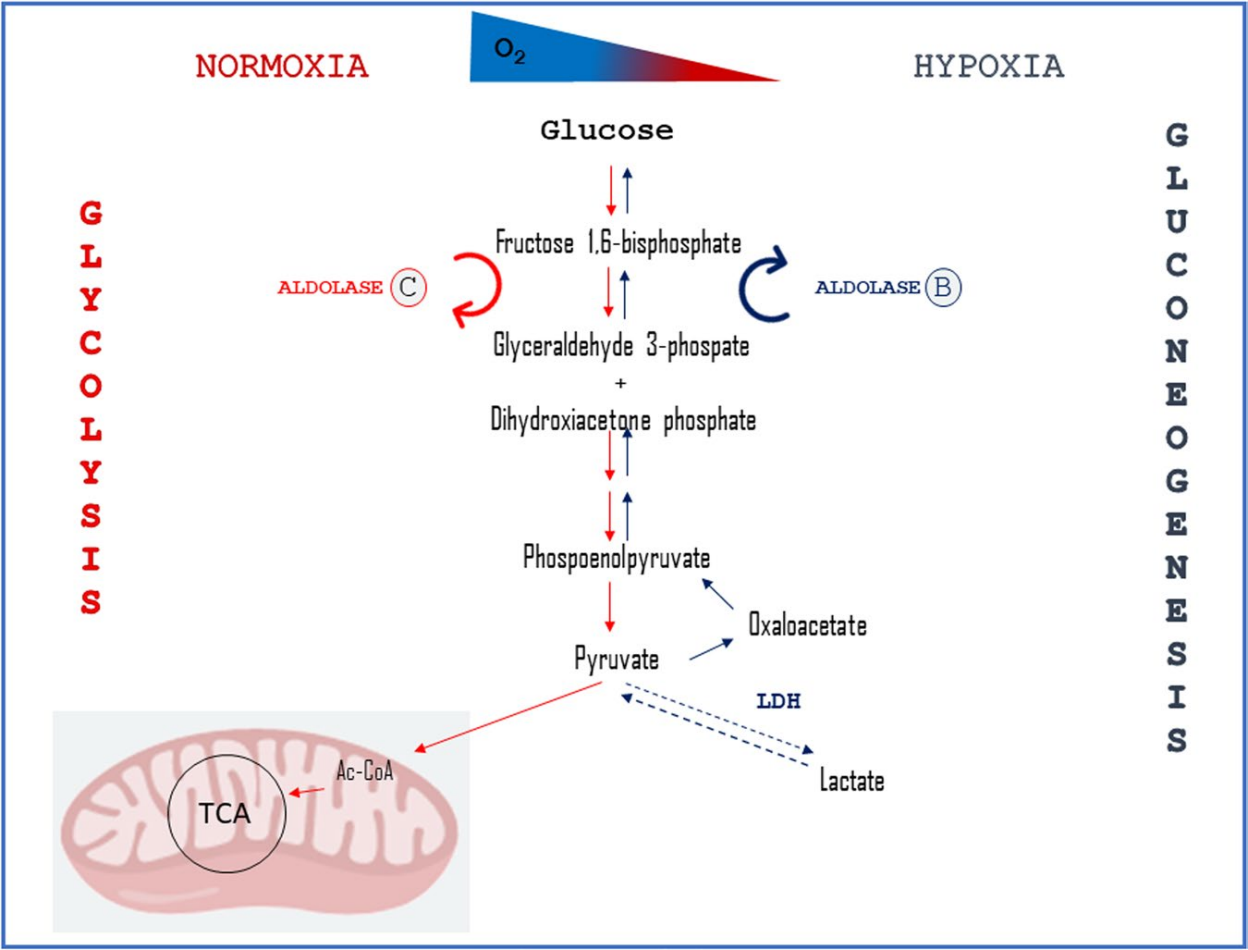
Model for alternative routes of pyruvate metabolism in the goldfish (*C. auratus*) heart. Pyruvate from glycolysis is either converted to acetyl-CoA (Ac-CoA) (under normoxia) or to lactate by the lactate dehydrogenase (LDH) (under hypoxia). As suggested by our analysis, under low oxygen levels, LDH activity is reduced (dashed arrows), and pyruvate may be converted in oxaloacetate and re-used in gluconeogenesis.

The ability of the hypoxic cyprinid fish to maintain a normal (*C. carassius*)^20^, or even enhanced (*C. auratus*)^21^ cardiac performance has been related to the capacity to cope with acidosis associated with anaerobic end-products accumulation (e.g. lactate)^51^. This may occur either through a metabolic removal of lactate and/or to the recruitment of pyruvate in alternative metabolic pathways (e.g. gluconeogenesis). Several evidences suggest that the goldfish, as well as its relative, the crucian carp, is able to reduce lactate to ethanol, an acid–base neutral molecule, easily excreted by the gills^52,53^. The production of ethanol exclusively occurs in the swimming muscle, which possesses the biochemical machinery to process not only the lactate generated within the muscle itself, but also that produced by other tissues such as brain, liver and heart and delivered to the muscle *via* the blood flow^54,55^. In the whole organism, this mechanism efficiently allows the conversion of waste lactate in a harmless anaerobic end product. However, it takes time, depending on the physiological circulatory interactions between organs and tissues. Moreover, it does not fully justify the increase of cardiac performance observed in the isolated and perfused goldfish heart in response to hypoxia^21^. Indeed, according to mammalian paradigms, in an isolated organ (i.e. a “closed system”), anaerobic lactate and H^+^ accumulation should lead to intracellular acidosis, impairment of myocardial contractility/relaxation and hence a dramatic decrease of the heart ability to pump blood^56,57^. In this context, and in agreement with the bioinformatics evidence of the interaction between lactate dehydrogenase A (ldha) and fructose-bisphosphate aldolase B (aldob), our biochemical assays allows to propose also a role of gluconeogenesis in the mechanisms involved in the response of the goldfish heart to hypoxia (Fig. 6). The hypoxia-induced activation of glucose re-synthesis, allowing a continuous recycle of pyruvate, contributes to prevent the production of deleterious wastes, thus assuring the maintenance of cardiac pumping ability.

## Conclusion

In this work, we perform a selective extraction of goldfish heart proteins followed by tandem mass spectrometric analysis. The observed protein composition proves that the adopted experimental condition is an effective alternative to the classic extraction methods to look at the most stable proteins. This approach led the identification of 12 most stable hypoxia regulated proteins. It represents the first experimental report of *in vitro* normoxic and hypoxic goldfish cardiac tissues, proving that aldolase B may be a novel hypoxic marker. The findings agree with the hypothesis that under conditions of reduced oxygen, a modulation of the aldolase enzyme isoforms allows the goldfish heart to recycle the first product of the anaerobic glycolysis, namely pyruvate. This may minimize deleterious waste accumulation, allowing, at the same time, to mitigate the negative consequences of a low hypoxia-dependent ATP production. Our data suggest that, in the goldfish heart, a highly coordinated regulatory system may fine-tune glycolytic vs gluconeogenic flux in relation to oxygen availability, thus contributing to hypoxia tolerance.

## Methods

### Animals

The study was conducted on 15 specimens of goldfish *C. auratus* (mass = 42 ± 2.3 g; mean ± SD), provided by a fish farm (COF SAS, Bologna, Italy). Animals were kept at room temperature (18–20 °C) for 7–10 days, and daily fed with commercial food. They were anesthetized with MS222 (tricaine methanesulfonate; 0.2 g/L) (Sigma–Aldrich, Italy), and then killed by cervical transection.

Hearts were dissected out and directed to the specific protocol^58^. Animal care and procedures were in accordance with the U.S. National Institutes of Health’s Guide for the Care and Use of Laboratory Animals (NIH Publication No. 85–23, revised 1996) and with the Italian law (DL 116, January 27, 1992), which did not require a specific authorization for the used species by an ethics committee.

### Isolated and *in vitro* perfused working heart preparations

The hearts, removed without the parietal pericardium and cannulated, were transferred to a perfusion chamber filled with Ringer’s solution and connected to a perfusion apparatus as previously described^59,60^. The hearts received Ringer’s solution from an input reservoir and pumped against an afterload pressure given by the height of an output reservoir. The Ringer composition was (in mmol/L) NaCl: 124.9, KCl: 2.49, MgSO_4_: 0.94, NaH_2_PO_4_: 1, glucose: 5, NaHCO_3_: 15 and CaCl_2_: 1.2. For normoxic experiments, the saline was equilibrated with a mixture of 99.5% O_2_ and 0.5% CO_2_. For hypoxic experiments, the ringer was equilibrated with a mixture of 10% O_2_, 0.5% CO_2_ and 89.5% N_2_^21^. Experiments were carried out at room temperature (18–20 °C). Hemodynamic parameters were obtained as previously described^61^. Briefly, measures were performed by using two MP-20D pressure transducers (Micron Instruments, Simi Valley, CA, USA) connected to a PowerLab data acquisition system and analyzed by using Chart software (ADInstruments, Basile, Italy). Pressures were corrected for cannula resistance. Cardiac output (CO) was collected over 1 min and weighed. Values were corrected for fluid density and expressed as volume measurements. Heart rate (HR) was obtained from pressure traces. Stroke volume (SV = CO/HR) and stroke work [SW; mJ/g; (afterload − preload) × SV/ventricle mass] were used as indexes of ventricular performance and systolic functionality, respectively.

Isolated and perfused goldfish hearts were allowed to maintain a spontaneous rhythm for up to 15–20 min. In all experiments, control conditions were a mean output pressure of about 1.5 kPa, with a CO set to 10–12 ml/min/kg body mass by appropriately adjusting the filling pressure^62^. The heart generated its own rhythm. Cardiac variables were measured every 10 min of perfusion for 90 min simultaneously during experiments. Hearts that did not stabilize within 20 min of perfusion were discarded. At the end of the experiment, hearts were immediately immersed in liquid nitrogen and stored at −80 °C for further analysis.

### SDS-PAGE

Hearts perfused in normoxic and hypoxic conditions were weighed, shredded, and equally divided. One half was homogenized in 0,05 M ammonium bicarbonate [pH 8.0; 1:10 (w/v)], the other one in RIPA buffer (Sigma Aldrich) [pH 8.0; 1:10 (w/v)], by using a glass potter homogenizing vessel with a Teflon pestle on ice. Homogenates were centrifuged at 1000 g for 10 min at 4 °C to remove tissue debris. After centrifugation the supernatant was collected and Bradford reagent was used to determine protein concentration according to the manufacturer (Sigma–Aldrich). Amounts of 4 µg protein of each tissue were separated on SDS/12% (w/v) polyacrylamide gel and then stained in blu comassie for mass spectrometry analysis.

### Mass spectrometry

Mass spectrometry analyses were performed using a 5800 MALDI-TOF-TOF Analyzer (AB SCIEX, Darmstadt, Germany), using a neodymium: yttrium-aluminum-garnet laser (349 nm), in reflectron positive mode with a mass accuracy of 5 ppm. The adopted experimental conditions were already reported^32^.Briefly, 4000 laser shots were typically accumulated with a laser pulse rate of 400 Hz in the MS mode, whereas in the MS/MS mode spectra up to 5000 laser shots were acquired and averaged with a pulse rate of 1000 Hz. MS/MS experiments were performed at a collision energy of 1 kV, ambient air was used as collision gas with medium pressure of 10^−6^ Torr.

### Linear MALDI

1 μL of sample solution (80 nmol/μL, normoxic and hypoxic conditions) was mixed with 2 μL of α-CHCA (5 mg/mL; H 2 O/CH 3 CN, 40:60 (v/v) with 0.3% TFA). A 1 μL portion of sample–matrix solution was spotted on a MALDI plate and dried at room temperature. Linear MALDI MS spectra were acquired averaging 4000 laser shots with a mass accuracy of 500 ppm in default calibration mode that was performed using the following set of standards: aldolase (rabbit, [M + H] + avg = 39905), BSA (bovin serum albumin [M + H] + avg = 66431) and IgG1 (murine myeloma [M + H] + avg = 148500)^33^.

### Mass spectrometry data processing

Protein identification was performed with the Protein Pilot 4.0 software program (AB SCIEX) using the Paragon (AB SCIEX) protein database search algorithm. The data analysis parameters were as follows: Sample type: Identification; Cys Alkylation: None; Digestion: Trypsin; Instrument: 5800; Special factors: Phosphorylation emphasis; Species: None; ID Focus: Biological modifications –Amino acid substitution; Database: uniprot-taxonomy_Actiinopterigji_7898.fasta; Search Effort: Thorough ID; FDR analysis: Yes; Detected Protein Threshold [Unused ProtScore (Conf)]: 1.5 (95.0%). After acquisition, spectra were handled using Data Explorer version 4.11 (AB Sciex). The MS/MS data were also processed to assign candidate peptides in the NCBI, SwissProt database using the MASCOT search program (http://www.matrixscience.com). The mass tolerance of the parent and fragments for MS/MS data search was set at 10 ppm and 0.20 Da, respectively. The query was made for “the ray-finned fishes (Actinopterygii)” taxonomy allowing 2 missed cleavage. A Peaklist of 50 ions of intensity higher 10% above the noise level were generically used for database search. The phosphorylation of serine and threonine was included in the variable modifications. Most of MS/MS spectra showed intense and well resolved ion signals. However, all spectra were manually checked^63,64^ to verify the validity of the MASCOT results.

### Functional and gene ontology (GO) analysis

The list of non-redundant protein IDs was subjected to PANTHER program (http://www.pantherdb.org/) to identifying protein class and biological process. The whole *Danio rerio* genome was selected as a reference set. A statistical significance of representation for the analysis is provided (Table 15S). Functional Annotation Clustering were obtained using DAVID software (http://david.abcc.ncifcrf.gov/). For that, all protein entries were processed indicating the corresponding zebrafish reference entry (ZIFN ID, https://www.uniprot.org/) UniProtKB (Tables 13S and 14S).

### Network analysis

Network analysis was performed submitting the orthologous *Danio rerio* gene IDs to the STRING (Search Tool for the Retrieval of Interacting Genes) software (v.11) (http://stringdb.org/)^65^. This is a large database of known and predicted protein interactions. Proteins were represented with nodes, direct (physical) interactions with continuous lines, while indirect (functional) interactions were presented by dashed lines. All the edges were supported by at least a reference from the literature or from canonical information stored in the STRING dataset. A confidence score was fixed to 0.7 (high confidence). Cluster networks were created using the MCL algorithm which is included in the STRING website and a value of 3 was selected for all the analyses.

### Biochemical assays: sample preparation

Perfused goldfish hearts were homogenized in ice-cold homogenization buffer (EDTA 0,025 M, NaCl 0,01 M, Tris-HCl 0,01 M pH 7.4, Triton 0,1%) containing a mixture of protease inhibitors (1 mmol/L aprotinin, 20 mmol/L phenylmethylsulfonyl fluoride, and 200 mmol/L sodium ortho-vanadate) and centrifuged at 10000 g for 10 min at 4 °C. Cytosolic fraction was collected and protein concentration determined with Bradford reagent.

### Pyruvate assay

Tissue pyruvate measurements were performed as described by Hegde *et al*.^66^. Briefly, cardiac homogenates were mixed with NADH and LDH reagent, and pyruvate concentration was enzymatically determined by monitoring spectrophotometrically the decrease in absorption (at 340 nm) due to the NADH-dependent reduction of pyruvate to lactate by LDH. The assay was done as follows: a reagent mixture consisting of 0.2 mM NADH in 0.1 M phosphate buffer, pH 7.5, and LDH (10 units) was prepared and the initial absorption (OD1) noted; 70 μl of the cardiac extract was then added to the reaction mixture (3 ml) and OD2 was read after 5 min. Pyruvate concentration, represented by the difference in absorption (OD1–OD2), was calculated with reference to the extinction coefficient of NADH and expressed as µmol/mg protein.

### Lactate dehydrogenase assay

LDH was determined by the method described by Lim *et al*. 1974^67^. For assay of lactate dehydrogenase activity, the oxidation of NADH was followed by measuring the rate of decrease in absorbance at 340 nm in a 3 ml reaction mixture containing potassium phosphate (0.1 M, pH 7.5), sodium pyruvate (0.66 mM) and NADH (0.2 mM), supplemented with 2 μL of tissue supernatant. Absorbance changes at 340 nm were measured with a Jasco V-530 UV/VIS spectrophotometer. All assays were performed at 25 °C. LDH activity was expressed as UI/mg protein.

### Statistics

Biochemical assay experiments were performed in triplicate; data were expressed as means ± SEM of absolute values from individual experiments. Statistical analysis was assessed by unpaired t-test (significance level at p < 0.05) by using GraphPad Prism software, version 5.02 (GraphPad Software Inc., San Diego, CA, USA, https://www.graphpad.com).

## Supplementary information

Supplementary Information

Supplementary Information
