# Supplementary material for: MS-based proteomic analysis of cardiac response to hypoxia in the goldfish (Carassius auratus)
Source: Sci Rep. 2019 Dec 12;9:18953. doi: 10.1038/s41598-019-55497-w (PMC6908699; doi:10.1038/s41598-019-55497-w)
Supplement: Supplementary file 1 — Supplementary Information [file 41598_2019_55497_MOESM1_ESM.docx]

**MS-based proteomic analysis of cardiac response to hypoxia in the goldfish (*Carassius* *auratus).***

Sandra Imbrogno^1^, Donatella Aiello^2^, Mariacristina Filice^1^, Serena Leo^1^, Rosa Mazza^1^, Maria Carmela Cerra^1^, Anna Napoli^2*^

^1^Dept of Biology, Ecology and Earth Sciences (BEST), University of Calabria, Arcavacata di Rende (CS), Italy;

^2^Department of Chemistry and Chemical Technologies, University of Calabria, Italy.

**Table 1S**

|  | **Myoglobin**  **B3CJI6_CARAU** | | | | | |
| --- | --- | --- | --- | --- | --- | --- |
|  | **Sequence** | **Modifications** | **Start-End** | **Δppm** | **Prec m/z** | **Theor m/z** |
| **1** | ADHELVLK | Protein Terminal Acetyl@N-term | 2-9 | 5.4 | 966.53 | 966.5255 |
| **2** | CWGVVEADFEGTGGEVLTR |  | 9-27 | 5.0 | 2024.95 | 2024.938 |
| **3** | LFKQHPETQK |  | 28-37 | 7.0 | 1255.63 | 1255.618 |
| **4** | LFKQHPETQKLFPK |  | 28-41 | 6.0 | 1740.99 | 1740.979 |
| **5** | QHPETQKLFPK |  | 31-41 | 6.0 | 1352.74 | 1352.732 |
| **6** | QHPETQKLFPK | Gln->pyroGlu@N-term | 32-42 | 5.0 | 1335.71 | 1335.706 |
| **7** | KLGELLKAR |  | 67-75 | 6.0 | 1026.67 | 1026.6628 |
| **8** | GDHAAILKPLATTHANK |  | 79-91 | 5.8 | 1757.98 | 1757.966 |
| **9** | HKIALNNFR |  | 92-100 | 6.4 | 1112.64 | 1112.632 |
| **10** | LITEVLVK |  | 101-108 | 6.7 | 914.60 | 914.5921 |
| **11** | AGLDAAGQTALR | Thr->Ser@9 | 115-126 | 5.5 | 1129.60 | 1129.596 |

|  |  |  | |  | |  | |  | |  |
| --- | --- | --- | --- | --- | --- | --- | --- | --- | --- | --- |
| **1** | M**ADHELVLKC** | | **WGVVEADFEG** | | **TGGEVLTRLF** | | **KQHPETQKLF** | | **PK**FVGIAQSD | |
| **51** | LAGNAAVNAH | | GATVLK**KLGE** | | **LLKARGDHAA** | | **ILKPLATTHA** | | **NKHKIALNNF** | |
| **101** | **RLITEVLVK**V | | MAEK**AGLDAA** | | **GQTALR**KVME | | AVIGDIDTYY | | KEFGFAG | |

**Sequence coverage 65%**

**Table 2S**

|  | **Hemoglobin subunit beta**  **HBB_CARAU; B0BL35_CARAU; Q1RM32_DANRE; Q98852_CYPCA; Q98851_CYPCA; P70073_CYPCA; O13140_CYPCA** | | | | | |
| --- | --- | --- | --- | --- | --- | --- |
|  | **Sequence** | **Modifications** | **Start-End** | **Δppm** | **Prec m/z** | **Theor m/z** |
| **1** | VEWTDAER |  | 1-8 | 6.0 | 1005.47 | 1005.4641 |
| **2** | SAIIGLWGK |  | 9-17 | 6.1 | 944.56 | 944.5569 |
| **3** | LNPDELGPQALAR |  | 18-30 | 6.5 | 1393.75 | 1393.7439 |
| **4** | YFATFGNLSSPAAIMGNPK | Oxidation (M) | 41-59 | 5.8 | 2001.99 | 2001.9744 |
| **5** | VAAHGRTVMGGLER |  | 60-73 | 5.9 | 1453.78 | 1453.7698 |
| **6** | ATYAPLSVMHSEK |  | 83-95 | 5.7 | 1433.72 | 1433.7099 |
| **7** | ATYAPLSVMHSEK | Oxidation (M) | 83-95 | 5.5 | 1449.71 | 1449.7048 |
| **8** | LHVDPDNFR |  | 96-104 | 6.2 | 1112.56 | 1112.5489 |
| **9** | FGPSGFNADVQEAWQK |  | 118-133 | 5.9 | 1780.84 | 1780.8294 |
| **10** | FLAVVVSALCR | Cys->Dha(C)@10 | 135-145 | 5.4 | 1143.70 | 1143.6890 |

TR|Q98852|Q98852_CYPCA M**VEWTDAERSAIIGLWGK**LNPDELGPQALARCLIVYPWTQR**YFASFGNLSSPAAIMGNPK** 60

TR|Q98851|Q98851_CYPCA M**VEWTDAERSAIIGLWGK**LNPDELGPQALARCLIVYPWTQR**YFASFGNLSSPAAIMGNPK** 60

TR|P70073|P70073_CYPCA M**VEWTDAERSAIIGLWGK**LNPDELGPQALARCLIVYPWTQR**YFASFGNLSSPAAIMGNPK** 60

TR|O13140|O13140_CYPCA M**VEWTDAERSAIIGLWGK**LNPDELGPQALARCLIVYPWTQR**YFASFGNLSSPAAIMGNPK** 60

**TR|B0BL35|B0BL35_CARAU** M**VEWTDAERSAIIGLWGK**LNPDELGPQALARCLIVYPWTQR**YFATFGNLSSPAAIMGNPK** 60

**SP|P02140|HBB_CARAU** -**VEWTDAERSAIIGLWGK**LNPDELGPQALARCLIVYPWTQR**YFATFGNLSSPAAIMGNPK** 59

TR|Q1RM32|Q1RM32_DANRE M**VEWTDAER**TAILGLWGKLNIEEIGPQALSRCLIVYPWTQR**YFATFGNLSSPAAIMGNPK** 60

********:**:******* :*:*****:**************:***************

TR|Q98852|Q98852_CYPCA **VAAHGRTVMGGLER**AIKNMDNIK**ATYAPLSVMHSEKLHVDPDNFR**LLADCITVCVAMKFG 120

TR|Q98851|Q98851_CYPCA **VAAHGRTVMGGLER**AIKNMDNIK**ATYAPLSVMHSEKLHVDPDNFR**LLADCITVCVAMK**FG** 120

TR|P70073|P70073_CYPCA **VAAHGRTVMGGLER**AIKNMDNIK**ATYAPLSVMHSEKLHVDPDNFR**LLADCITVCAAMKFG 120

TR|O13140|O13140_CYPCA **VAAHGRTVMGGLER**AIKNMDNIK**ATYAPLSVMHSEKLHVDPDNFR**LLADCITVCAAMK**FG** 120

**TR|B0BL35|B0BL35_CARAU** **VAAHGRTVMGGLER**AIKNMDNIK**ATYAPLSVMHSEKLHVDPDNFR**LLADCITVCAAMK**FG** 120

**SP|P02140|HBB_CARAU** **VAAHGRTVMGGLER**AIKNMDNIK**ATYAPLSVMHSEKLHVDPDNFR**LLADCITVCAAMK**FG** 119

TR|Q1RM32|Q1RM32_DANRE **VAAHGRTVMGGLER**AIKNMDNVKNTYAALSVMHSEK**LHVDPDNFR**LLADCITVCAAMKFG 120

*********************:* *** **************************.*****

TR|Q98852|Q98852_CYPCA PSGFNPDVQEAWQKFLCVVVSALCRQYH 148

TR|Q98851|Q98851_CYPCA **PSGFNADVQEAWQK**FLCVVVSALCRQYH 148

TR|P70073|P70073_CYPCA PSGFSPNVQEAWQKFLSVVVSALCRQYH 148

TR|O13140|O13140_CYPCA **PSGFNADVQEAWQK**FLCVVVSALCRQYH 148

**TR|B0BL35|B0BL35_CARAU** **PSGFNADVQEAWQK**FLSVVVSALCRQYH 148

**SP|P02140|HBB_CARAU** **PSGFNADVQEAWQK**FLSVVVSALCRQYH 147

TR|Q1RM32|Q1RM32_DANRE QAGFNADVQEAWQK**FLAVVVSALCR**QYH 148

:**. :*********.***********

**HBB_CARAU Sequence coverage 60%**

**Table 3S**

|  | **Glyceraldehyde-3-phosphate dehydrogenase**  **G3P_DANRE; Q4VSK0_CARAU; S4SRW0_9TELE; J9TGM4_CATCA; I6YYG2_CYPCA; Q2A665_SALSA** | | | | | |
| --- | --- | --- | --- | --- | --- | --- |
|  | **Sequence** | **Modifications** | **Start-End** | **Δppm** | **Prec m/z** | **Theor m/z** |
| **1** | MVKVGINGFGRIGR |  | 1-14 | 6.0 | 1504.87 | 1503.8582 |
| **2** | VKVGINGFGR |  | 2-11 | 6.5 | 1046.62 | 1046.6111 |
| **3** | VGINGFGRIGRLVTRAAFLTK |  | 4-24 | 5.7 | 2246.34 | 2246.3249 |
| **4** | LVTRAAFLTKK |  | 15-25 | 5.5 | 1247.79 | 1247.7839 |
| **5** | YKGEVKAEGGK |  | 54-64 | 6.2 | 1165.63 | 1165.6217 |
| **6** | GEVKAEGGKLVIDGHAITVYSER |  | 56-78 | 5.9 | 2428.30 | 2428.2836 |
| **7** | YDNSLTVVSNASCTTNCLAPLAK |  | 138-160 | 5.4 | 2385.16 | 3285.1430 |
| **8** | TVDGPSGKLWR |  | 185-195 | 5.9 | 1215.66 | 1215.6486 |
| **9** | LWRDGRGASQNIIPASTGAAK |  | 193-213 | 5.8 | 2168.17 | 2169.1528 |
| **10** | DGRGASQNIIPASTGAAK |  | 196-213 | 5.9 | 1713.90 | 1713.8883 |
| **11** | GASQNIIPASTGAAK |  | 199-213 | 5.7 | 1385.75 | 1385.7388 |
| **12** | GASQNIIPASTGAAKAVGK |  | 199-217 | 6.1 | 1741.97 | 1740.9608 |
| **13** | VPTPNVSVVDLTVR |  | 233-246 | 6.3 | 1495.86 | 1495.8484 |
| **14** | LEKPAKYDEIK |  | 247-257 | 5.5 | 1333.740 | 1333.7367 |
| **15** | YDEIKKVVKAAADGPMK |  | 253-269 | 6.2 | 1863.02 | 1863.0049 |
| **16** | GILGYTEHQVVSTDFNGDCR |  | 270-289 | 5.9 | 2211.33 | 2211.0140 |
| **17** | GILGYIEDQVVSSDFNGDVR | Leu->Thr@3 | 270-289 | 6.0 | 2171.04 | 2171.0256 |
| **18** | GILGYTEDQVVSTDFNGDVR | Thr->Ser@13 | 270-289 | 5.4 | 2199.07 | 2199.0569 |
| **19** | SSIFDAGAGIALNDHFVK |  | 290-307 | 6.6 | 1861.96 | 1861.9448 |
| **20** | LVTWYDNEFGYSNR |  | 308-321 | 6.2 | 1763.81 | 1763.8029 |
| **21** | VCDLMAHMASKE |  | 322-333 | 5.4 | 1334.60 | 1334.5907 |

TR|Q4VSK0|Q4VSK0_CARAU -------GFGRIGRLVLRACL--QKGIKVTAINDPFIDLKYMVYMFKYDSTHGRYKGDVH 51

TR|S4SRW0|S4SRW0_9TELE **MVKVGINGFGRIGR**LVTRAAFQSK-KVEIVAINDPFIDLEYMVYMFKYDSTHGK**YKGEVK** 59

TR|J9TGM4|J9TGM4_LABCA ------------------------------AINDPFIDLEYMVYMFKYDSTHGK**YKGEVK** 30

SP|Q5XJ10|G3P_DANRE **MVKVGINGFGRIGRLVTRAAFLTK**-**K**VEIVAINDPFIDLDYMVYMFQYDSTHGK**YKGEVK** 59

TR|I6YYG2|I6YYG2_CYPCA ------------------------------------------------------------

TR|Q2A665|Q2A665_SALSA MVKVGVNGFGRIGRLVTRAAFHSKKGVEIVAINDPFIDLDYMVYMFKYDSTHGRFHGEVK 60

TR|Q4VSK0|Q4VSK0_CARAU LEDGKMIVDGQAISVFQCMKPAEIPWGDAGALYVVESTGVFLSIDKASSHIQGGAKRVVV 111

TR|S4SRW0|S4SRW0_9TELE **AEGGKLVIDGHAITVSSER**DPANIKWGDAGATYVVESTGVFTTIEKASAHLKGGAKRVII 119

TR|J9TGM4|J9TGM4_LABCA **AEGGK**LVIDGQAITVFSERDPSNIKWGDAGAQYVVESTGVFTTIEKASAHLKGGAKRVII 90

SP|Q5XJ10|G3P_DANRE **AEGGKLVIDGHAITVYSER**DPANIKWGDAGATYVVESTGVFTTIEKASAHIKGGAKRVII 119

TR|I6YYG2|I6YYG2_CYPCA ------------------------------------------------------------

TR|Q2A665|Q2A665_SALSA AEGGKLVIDGHKITVFHERDPANIKWGDAGATYVVESTGVFTTIEKASTHLEGGAKRVVI 120

TR|Q4VSK0|Q4VSK0_CARAU SAPSPDAPMFVMGVNQDKYDPSSMTIVSNASCTTNCLAPLAKVIQDNFGIEEALKTTVHA 171

TR|S4SRW0|S4SRW0_9TELE SAPSADAPMFVMGVNHEKYDNS-LKVVSNASCTTNCLAPLAKVINDNFVIIEGLMSTVHA 178

TR|J9TGM4|J9TGM4_LABCA SAPSADAPMFVMGVNHEKYDNS-LKVVSNASCTTNCLAPLAKVIHDNFVIVEGLMSTVHA 149

SP|Q5XJ10|G3P_DANRE SAPSADAPMFVMGVNHEK**YDNS-LTVVSNASCTTNCLAPLAK**VINDNFVIVEGLMSTVHA 178

TR|I6YYG2|I6YYG2_CYPCA ------------------------------------------------------------

TR|Q2A665|Q2A665_SALSA SAPSADAPMFVMGVNHEKYDNS-LKVVSNASCTTNCLAPLAKVIHDNYHIIEGLMSTVHG 179

TR|Q4VSK0|Q4VSK0_CARAU YTATQKTVDGPSAKAWRDGRGAHQNIIPASTGAAKAVGK--------------------- 210

TR|S4SRW0|S4SRW0_9TELE ITATQK**TVDGPSGKLWRDGRGASQNIIPASTGAAK**AVGKVIPELNGKITGMAFR**VPTPNV** 238

TR|J9TGM4|J9TGM4_LABCA ITATQK**TVDGPSGKLWRDGRGASQNIIPASTGAAKAVGK**VIPELNGKLTGMAFR**VPTPNV** 209

SP|Q5XJ10|G3P_DANRE ITATQK**TVDGPSGKLWRDGRGASQNIIPASTGAAKAVGK**VIPELNGKLTGMAFR**VPTPNV** 238

TR|I6YYG2|I6YYG2_CYPCA -----K**TVDGPSGKLWRDGRGASQNIIPASTGAAKAVGK**VIPELNGKLTGMAFR**VPTPNV** 55

TR|Q2A665|Q2A665_SALSA VTATQK**TVDGPSGKLWRDGRGASQNIIPASTGAAKAVGK**VIPELNGKITGMAFR**VPTPNV** 239

*******.* ******* ****************

TR|Q4VSK0|Q4VSK0_CARAU ------------------------------------------------------------

TR|S4SRW0|S4SRW0_9TELE **SVVDLTVRLEKPAKYDEIKKVVKAAADGPMKGILGYIEDQVVSSDFNGDVRSSIFDAGAG** 298

TR|J9TGM4|J9TGM4_LABCA **SVVDLTVRLEKPAKYDEIKKVVKAAADGPMKGILGYTEDQVVSTDFNGDVRSSIFDAGAG** 269

SP|Q5XJ10|G3P_DANRE **SVVDLTVRLEKPAKYDEIKKVVKAAADGPMKGILGYTEHQVVSTDFNGDCRSSIFDAGAG** 298

TR|I6YYG2|I6YYG2_CYPCA **SVVDLTVR**LEKPAKYDDIKKVVKAAADGPMK**GILGYTEDQVVSTDFNGDVRSSIFDAGAG** 115

TR|Q2A665|Q2A665_SALSA **SVVDLTVR**LEKPASYDAIKKVVKAAADGPMKGILGYTEQQVVSSDFNGDTH**SSIFDAGAG** 299

TR|Q4VSK0|Q4VSK0_CARAU -----------------------------------

TR|S4SRW0|S4SRW0_9TELE **IALNDHFVKLVTWYDNEYGYSNR**VCDLMAHMSTKE 333

TR|J9TGM4|J9TGM4_LABCA **IALNDHFVKLVTWYDNEFGYSNR**VCDLMAHMASKE 304

SP|Q5XJ10|G3P_DANRE **IALNDHFVKLVTWYDNEFGYSNRVCDLMAHMASKE** 333

TR|I6YYG2|I6YYG2_CYPCA **IALNDHFVK**L------------------------- 125

TR|Q2A665|Q2A665_SALSA **IALNDHFVKLVTWYDNEFGYSNR**VIDLMAHMATKE 334

**G3P_DANRE Sequence coverage 62%**

**Table 4S**

|  | **L-lactate dehydrogenase A chain**  **LDHA_FUNHE** | | | | | |
| --- | --- | --- | --- | --- | --- | --- |
|  | **Sequence** | **Modifications** | **Start-End** | **Δppm** | **Prec m/z** | **Theor m/z** |
| **1** | MSTQEKLISHVMK | Oxidation (M) | 1-13 | 6.0 | 1547.80 | 1547.7925 |
| **2** | STQEKLISHVMKEEPVGCR | Oxidation (M) | 2-20 | 6.3 | 2187.10 | 2187.0902 |
| **3** | LISHVMKEEPVGCRNK |  | 7-22 | 6.5 | 1839.97 | 1839.9573 |
| **4** | EEPVGCRNK |  | 14-22 | 5.8 | 1031.50 | 1031.4944 |
| **5** | NKVTVVGVGMVGMASAISVLLK |  | 21-42 | 5.6 | 2173.26 | 2173.2452 |
| **6** | DLCDELALVDVMEDK |  | 43-57 | 5.7 | 1707.79 | 1707.7821 |
| **7** | LKGEAMDLQHGALFLK | Oxidation (M) | 58-73 | 5.5 | 1786.96 | 1786.9525 |
| **8** | THKIVADK |  | 74-81 | 5.2 | 911.54 | 911.5314 |
| **9** | VVVVTAGARQQEGESR |  | 91-106 | 5.9 | 1685.90 | 1685.8934 |
| **10** | QQEGESRLNLVQR |  | 100-112 | 5.4 | 1556.82 | 1556.8144 |
| **11** | LNLVQRNVNIFKFIIPNIVK |  | 107-126 | 6.1 | 2382.45 | 2382.4389 |
| **12** | LSGFPRHR |  | 150-157 | 6.2 | 969.54 | 969.5382 |
| **13** | HRVIGSGTNLDSAR |  | 156-169 | 5.8 | 1482.79 | 1482.7777 |
| **14** | VIGSGTNLDSAR |  | 158-169 | 5.7 | 1189.62 | 1189.6177 |
| **15** | FRHLMGEK |  | 170-177 | 6.3 | 1017.54 | 1017.5304 |
| **16** | ELHKKVVDGAYEVIK |  | 229-243 | 5.5 | 1727.98 | 1727.9695 |
| **17** | VVDGAYEVIK |  | 234-243 | 5.3 | 1092.60 | 1092.5941 |
| **18** | VVDGAYEVIKLK |  | 234-245 | 6.4 | 1333.78 | 1333.7731 |
| **19** | GYTSWAIGMSVADLVESIVKNLHK |  | 246-269 | 5.9 | 2618.38 | 2618.3652 |
| **20** | QLVKSAETLWGVQK |  | 315-328 | 5.7 | 1586.90 | 1586.8906 |
| **21** | SAETLWGVQK |  | 319-328 | 5.9 | 1118.59 | 1118.5846 |

SP|Q92055|LDHA_FUNHE -**MSTQEKLISHVMKEEPVGCRNKVTVVGVGMVGMASAISVLLKDLCDELALVDVMEDKLK** 59

SP|Q92055|LDHA_FUNHE **GEAMDLQHGALFLKTHKIVADK**DYSVTANSK**VVVVTAGARQQEGESRLNLVQRNVNIFKF** 119

SP|Q92055|LDHA_FUNHE **IIPNIVK**YSPNCILLVVSNPVDILTYVAWK**LSGFPRHRVIGSGTNLDSARFRHLMGEK**FH 179

SP|Q92055|LDHA_FUNHE LHPSSCHGWIVGEHGDSSVAVWSGVNIAGVSLQTLNPNMGADGDSENWK**ELHKKVVDGAY** 239

SP|Q92055|LDHA_FUNHE **EVIKLKGYTSWAIGMSVADLVESIVKNLHK**VHPVSTLVQGMHGVKDEVFLSIPCVLGNSG 299

SP|Q92055|LDHA_FUNHE LTDVIHMTLKPEEEK**QLVKSAETLWGVQK**ELTL- 332

**Sequence coverage 60%**

**Table 5S**

|  | **Fructose-bisphosphate aldolase B**  **ALDOB_DANRE** | | | | | |
| --- | --- | --- | --- | --- | --- | --- |
|  | **Sequence** | **Modifications** | **Start-End** | **Δppm** | **Prec m/z** | **Theor m/z** |
| **1** | FQKINVENTEENR |  | 44-56 | 5.6 | 1620.81 | 1620.7982 |
| **2** | INVENTEENRR | Phospho (ST) | 47-57 | 5.7 | 1453.65 | 1453.6436 |
| **3** | SDKGVLFPKVIK |  | 88-99 | 5.9 | 1330.82 | 1330.8098 |
| **4** | DKGIVVGIK |  | 100-108 | 5.5 | 928.59 | 928.5831 |
| **5** | CAQYKKDGCDFAK |  | 135-147 | 5.2 | 1476.67 | 1476.6615 |
| **6** | YASICQQNGLVPIVEPEILPDGDHDLK |  | 174-200 | 5.7 | 2963.50 | 2963.4824 |
| **7** | QCQYATEKVLAAVYK |  | 201-215 | 5.9 | 1714.89 | 1714.8838 |
| **8** | ALSDHHVYLEGTLLK |  | 216-230 | 5.4 | 1695.92 | 1695.9070 |
| **9** | PNMVTAGHSCTK |  | 231-242 | 6.1 | 1245.58 | 1245.5720 |
| **10** | KYTPLEVAMATVTALR |  | 243-258 | 6.2 | 1763.98 | 1763.9729 |
| **11** | KYTPLEVAMATVTALRR | Phospho (ST) | 243-259 | 5.8 | 2000.05 | 2000.0404 |
| **12** | YTPLEVAMATVTALRR |  | 244-259 | 5.7 | 1791.99 | 1791.9791 |
| **13** | LSFSYGR | Phospho (ST) | 298-304 | 6.3 | 909.39 | 909.3871 |
| **14** | ALQASALSAWK |  | 305-315 | 5.9 | 1145.64 | 1145.6319 |
| **15** | ALQASALSAWKGQAANK |  | 305-321 | 5.8 | 1714.93 | 1714.9240 |
| **16** | GQAANKK |  | 316-322 | 6.2 | 716.41 | 716.4055 |
| **17** | ASQDAFVT |  | 323-331 | 5.5 | 838.40 | 838.3947 |
| **18** | AKINSLASKGEYK |  | 332-344 | 5.3 | 1408.79 | 1408.7799 |

|  |  |  |  |  |  |
| --- | --- | --- | --- | --- | --- |
| **1** | MTHQFPALST | EQKKELATIA | ERIVAPGKGI | LAADESTGTM | AKR**FQKINVE** |
| **51** | **NTEENRR**SFR | DLLFSVDDSI | SESIGGVILF | HETLYQK**SDK** | **GVLFPKVIKD** |
| **101** | **KGIVVGIK**VD | KGTAGLAGTD | GETTTQGLDG | LSER**CAQYKK** | **DGCDFAK**WRC |
| **151** | VLKISDSCPS | ALGIAENANV | LAR**YASICQQ** | **NGLVPIVEPE** | **ILPDGDHDLK** |
| **201** | **QCQYATEKVL** | **AAVYKALSDH** | **HVYLEGTLLK** | **PNMVTAGHSC** | **TKKYTPLEVA** |
| **251** | **MATVTALRR**T | VPAAVPGICF | LSGGQSEEEA | SLNLNAMNQL | SLHRPWK**LSF** |
| **301** | **SYGRALQASA** | **LSAWKGQAAN** | **KKASQDAFVT** | **RAKINSLASK** | **GEYK**PSGQAG |
| **351** | QASTQSLFTA | SYTY |  |  |  |

**Sequence coverage 68%**

**Table 6S**

|  | **Fructose-bisphosphate aldolase C**  **ALDOC_CARAU** | | | | | |
| --- | --- | --- | --- | --- | --- | --- |
|  | **Sequence** | **Modifications** | **Start-End** | **Δppm** | **Prec m/z** | **Theor m/z** |
| **1** | ELQDIAQR |  | 15-22 | 6.3 | 972.52 | 972.5114 |
| **2** | RLNPIGVENTEENRR |  | 43-57 | 6.5 | 1796.95 | 1796.9367 |
| **3** | QLLFTADERMDK |  | 61-72 | 6.4 | 1466.74 | 1466.7313 |
| **4** | MIKDRGIVVGIK |  | 97-108 | 5.9 | 1328.82 | 1328.8088 |
| **5** | LNPIGVENTEENR |  | 44-56 | 5.8 | 1484.74 | 1484.7345 |
| **6** | LNPIGVENTEENRR |  | 44-57 | 5.6 | 1640.84 | 1640.8356 |
| **7** | CAQYKKDGADFAK |  | 135-147 | 5.7 | 1444.70 | 1444.6894 |
| **8** | DGADFAKWR |  | 141-149 | 5.5 | 1065.52 | 1065.5174 |
| **9** | ISETSPSELAIMENANVLAR |  | 154-173 | 5.7 | 2145.10 | 2145.0861 |
| **10** | RCQYVTEK |  | 201-208 | 5.9 | 1026.51 | 1026.5042 |
| **11** | VLAACYK |  | 209-215 | 5.8 | 767.42 | 767.4126 |
| **12** | ALSDHHVYLEGTLLK |  | 216-230 | 5.2 | 1695.92 | 1695.9070 |
| **13** | PNMVTAGHSCPTK |  | 231-243 | 6.2 | 1342.63 | 1342.6248 |
| **14** | ALQASALSAWRGVKENEK |  | 305-322 | 5.9 | 1958.06 | 1958.0459 |
| **15** | GVKENEKAATEEFLKR |  | 316-331 | 5.4 | 1848.99 | 1848.9819 |
| **16** | AATEEFLKRGK |  | 323-331 | 6.1 | 1064.58 | 1064.5740 |
| **17** | RAEANGLAAQGK |  | 332-342 | 5.7 | 1185.64 | 1185.6340 |
| **18** | YVSSGMDGSAGQSLYVANHAY |  | 343-363 | 6.2 | 2176.97 | 2176.9609 |

|  |  |  |  |  |  |
| --- | --- | --- | --- | --- | --- |
| **1** | MTHQYPALTT | EQKR**ELQDIA** | **QR**IVAPGKGI | LAADESTGSM | AK**RLNPIGVE** |
| **51** | **NTEENRR**LYR | **QLLFTADERM** | **DK**CIGGVIFF | HETLYQKADD | GTPFAK**MIKD** |
| **101** | **RGIVVGIK**VD | KGVVPLAGTN | GETTTQGLDG | LSER**CAQYKK** | **DGADFAKWR**S |
| **151** | VLK**ISETSPS** | **ELAIMENANV** | **LAR**YASICQQ | NGIVPIVEPE | ILPDGDHDLK |
| **201** | **RCQYVTEKVL** | **AACYKALSDH** | **HVYLEGTLLK** | **PNMVTAGHSC** | **PTK**FSNQEIA |
| **251** | MATVTALRRT | VPPAVTGVTF | LSGGQSEEEA | SINLNAINNC | PLTKPWALTF |
| **301** | SYGR**ALQASA** | **LSAWRGVKEN** | **EKAATEEFLK** | **RAEANGLAAQ** | **GKYVSSGMDG** |
| **351** | **SAGQSLYVAN** | **HAY** |  |  |  |

**Sequence coverage 51%**

**Table 7S**

|  | **Beta-enolase**  **ENOB_SALSA** | | | | | |
| --- | --- | --- | --- | --- | --- | --- |
|  | **Sequence** | **Modifications** | **Start-End** | **Δppm** | **Prec m/z** | **Theor m/z** |
| **1** | IHAREILDSR |  | 6-15 | 5.7 | 1209.68 | 1209.6704 |
| **2** | EILDSRGNPTVEVDLYTAK |  | 10-28 | 6.3 | 2120.10 | 2120.0875 |
| **3** | GNPTVEVDLYTAK |  | 16-28 | 5.5 | 1406.72 | 1406.7167 |
| **4** | GNPTVEVDLYTAKGR |  | 16-30 | 5.3 | 1619.85 | 1619.8393 |
| **5** | AAVPSGASTGVHEALELR |  | 33-50 | 6.4 | 1764.94 | 1764.9244 |
| **6** | LIEKKFSVVDQEK |  | 77-89 | 5.9 | 1562.89 | 1562.8793 |
| **7** | AGAAEKGVPLYR |  | 121-132 | 5.7 | 1231.69 | 1231.6798 |
| **8** | IIIGMDVAASEFYKAGK |  | 240-256 | 5.9 | 1812.97 | 1812.9569 |
| **9** | YITGDQLGDLYK |  | 270-281 | 5.8 | 1385.70 | 1385.6952 |
| **10** | GYPVQSIEDPFDQDDWAAWTK |  | 286-306 | 5.9 | 2468.12 | 2468.1046 |
| **11** | IQQAVEKK |  | 328-335 | 6.2 | 943.56 | 943.5576 |
| **12** | VNQIGSVTESIKACK |  | 344-358 | 5.7 | 1576.85 | 1576.8368 |
| **13** | LAQSNGWGVMVSHR |  | 359-372 | 5.7 | 1541.77 | 1541.7647 |
| **14** | SGETEDTFIADLVVGLCTGQIK |  | 373-394 | 5.9 | 2296.15 | 2296.1382 |
| **15** | TGAPCRSER |  | 395-403 | 5.8 | 976.47 | 976.4634 |
| **16** | LAKYNQLMR | Oxidation (M) | 404-412 | 5.5 | 1152.63 | 1152.6199 |
| **17** | IEEELGAKAK |  | 413-422 | 5.9 | 1087.61 | 1087.5999 |
| **18** | AKFAGKDYRHPK |  | 421-432 | 6.2 | 1471.78 | 1471.7704 |

| **1** | MSITK**IHARE** | **ILDSRGNPTV** | **EVDLYTAKGR** | FR**AAVPSGAS** | **TGVHEALELR** |
| --- | --- | --- | --- | --- | --- |
| **51** | DGDKSRYLGK | GTVKAVDHVN | KDIAAK**LIEK** | **KFSVVDQEK**I | DHFMLELDGT |
| **101** | ENKSKFGANA | ILGVSLAVCK | **AGAAEKGVPL** | **YR**HIADLAGH | KDVILPCPAF |
| **151** | NVINGGSHAG | NKLAMQEFMI | LPIGASNFHE | AMRIGAEVYH | NLKNVIKAKY |
| **201** | GKDATNVGDE | GGFAPNILEN | NEALELLKTA | IEKAGYPDK**I** | **IIGMDVAASE** |
| **251** | **FYKAGK**YDLD | FKSPDDPAR**Y** | **ITGDQLGDLY** | **K**SFIK**GYPVQ** | **SIEDPFDQDD** |
| **301** | **WAAWTK**FTAA | VDIQVVGDDL | TVTNPKR**IQQ** | **AVEKK**ACNCL | LLK**VNQIGSV** |
| **351** | **TESIKACKLA** | **QSNGWGVMVS** | **HRSGETEDTF** | **IADLVVGLCT** | **GQIKTGAPCR** |
| **401** | **SERLAKYNQL** | **MRIEEELGAK** | **AKFAGKDYRH** | **PK**IN |  |

**Sequence coverage 49%**

**Table 8S**

|  | **V-type proton ATPase subunit C 1-A**  **VTC1A_DANRE** | | | | | |
| --- | --- | --- | --- | --- | --- | --- |
|  | **Sequence** | **Modifications** | **Start-End** | **Δppm** | **Prec m/z** | **Theor m/z** |
| **1** | LMTATTRTNNLSTNNK |  | 22-37 | 6.0 | 1779.91 | 1779.9023 |
| **2** | TNNLSTNNKFNIPDLK |  | 29-44 | 6.3 | 1832.96 | 1832.9506 |
| **3** | VGTLDVLVGLSDELAK |  | 45-60 | 6.5 | 1628.92 | 1628.9110 |
| **4** | KVAQYMADVLEDSR |  | 71-84 | 5.8 | 1624.81 | 1624.8004 |
| **5** | KVAQYMADVLEDSRDK |  | 71-86 | 5.6 | 1868.93 | 1867.9224 |
| **6** | FQWDMAK |  | 105-111 | 5.7 | 925.43 | 925.4242 |
| **7** | FQWDMAKYPIK |  | 105-115 | 5.5 | 1426.73 | 1426.7193 |
| **8** | YPIKQSLKNISEIISK |  | 112-127 | 5.2 | 1861.09 | 1861.0799 |
| **9** | QSLKNISEIISK |  | 116-127 | 5.9 | 13659.79 | 1359.7847 |
| **10** | NISEIISK |  | 120-127 | 5.4 | 903.52 | 903.5151 |
| **11** | ARASAYNNLK |  | 138-147 | 6.1 | 1107.60 | 1107.5911 |
| **12** | ASAYNNLKGNLQNLER |  | 140-155 | 6.2 | 1804.94 | 1804.9306 |
| **13** | NAGSLLTR | Phospho (ST) | 157-164 | 5.8 | 911.44 | 911.4352 |
| **14** | NAGSLLTRSLADIVKK |  | 157-172 | 5.7 | 1686.00 | 1685.9914 |
| **15** | SLADIVKK | Phospho (ST) | 165-172 | 6.3 | 953.51 | 953.5072 |
| **16** | TYETLAEMVVPR |  | 200-211 | 5.5 | 1408.72 | 1408.7146 |
| **17** | KAIDDFRHKAR |  | 232-242 | 5.3 | 1356.75 | 1356.7500 |
| **18** | ADKEEMTRLSTDKK |  | 260-273 | 6.4 | 1651.84 | 1651.8325 |
| **19** | EEMTRLSTDK |  | 263-272 | 5.9 | 1209.59 | 1209.5785 |
| **20** | EEMTRLSTDKK |  | 263-273 | 5.7 | 1337.68 | 1337.6735 |
| **21** | KQFGPLVR |  | 274-281 | 5.9 | 944.57 | 944.5681 |
| **22** | KQFGPLVRWLK |  | 274-284 | 5.8 | 1371.83 | 1371.8265 |
| **23** | YGLPVNFQAMLLQPNKKNMK |  | 310-329 | 6.2 | 2335.86 | 2335.8410 |
| **24** | KNMKKLR |  | 326-332 | 5.7 | 917.58 | 917.5719 |
| **25** | NMKKLREVLYDLYK |  | 327-340 | 5.8 | 1813.02 | 1813.0046 |

| **1** | MTEFWLISAP | GEKTCQQTWD | K**LMTATTRTN** | **NLSTNNKFNI** | **PDLKVGTLDV** |
| --- | --- | --- | --- | --- | --- |
| **51** | **LVGLSDELAK** | LDAFVESVVK | **KVAQYMADVL** | **EDSRDK**VQEN | LLANGVDLVT |
| **101** | YVTR**FQWDMA** | **KYPIKQSLKN** | **ISEIISK**QVS | QIDNDLK**ARA** | **SAYNNLKGNL** |
| **151** | **QNLER**K**NAGS** | **LLTRSLADIV** | **KK**DDFVLDSE | YLITLLVVVP | KTNYTDWQR**T** |
| **201** | **YETLAEMVVP** | **R**STNLLFEDH | DSGLFTVTLF | R**KAIDDFRHK** | **AR**ENKFTVRD |
| **251** | FQYNEEEMK**A** | **DKEEMTRLST** | **DKKKQFGPLV** | **RWLK**VNFSEA | FIAWVHIKAL |
| **301** | RVFVESVLR**Y** | **GLPVNFQAML** | **LQPNKKNMKK** | **LREVLYDLYK** | HLDSSAAAII |
| **351** | DQSAMDIPGL | NLSQQEYYPY | VYYKIDCNLL | DFK |  |

**Sequence coverage 52%**

**Table 9S**

|  | **Actin, alpha skeletal muscle**  **ACTS_CARAU** | | | | | |
| --- | --- | --- | --- | --- | --- | --- |
|  | **Sequence** | **Modifications** | **Start-End** | **Δppm** | **Prec m/z** | **Theor m/z** |
| **1** | CDDEEVTALVCDNGSGLVK |  | 2-20 | 6.0 | 1966.89 | 1966.8738 |
| **2** | CDDEEVTALVCDNGSGLVKAGFAGDDAPR |  | 2-30 | 6.3 | 2924.32 | 2924.3042 |
| **3** | AGFAGDDAPR |  | 21-30 | 5.9 | 977.45 | 977.4488 |
| **4** | AVFPSIVGRPR |  | 31-41 | 5.8 | 1198.71 | 1198.7060 |
| **5** | HQGVMVGMGQKDSYVGDEAQSK |  | 42-63 | 5.6 | 2351.09 | 2351.0760 |
| **6** | HQGVMVGMGQKDSYVGDEAQSKR |  | 42-64 | 5.7 | 2507.19 | 2507.1771 |
| **7** | DSYVGDEAQSKR |  | 53-64 | 5.4 | 1354.63 | 1354.6239 |
| **8** | DSYVGDEAQSKRGILTLK |  | 53-70 | 5.2 | 1980.05 | 1980.0402 |
| **9** | GILTLKYPIEHGIITNWDDMEK |  | 65-86 | 5.9 | 2586.34 | 2586.3277 |
| **10** | YPIEHGIITNWDDMEK |  | 71-86 | 5.5 | 1960.92 | 1960.9115 |
| **11** | IWHHTFYNELR |  | 87-97 | 6.1 | 1515.76 | 1515.7497 |
| **12** | VAPEEHPTLLTEAPLNPK |  | 98-115 | 6.2 | 1956.06 | 1956.0442 |
| **13** | VAPEEHPTLLTEAPLNPKANREK |  | 98-120 | 5.8 | 2554.38 | 2554.3629 |
| **14** | DLTDYLMKILTER | Oxidation (M) | 186-198 | 5.7 | 1626.85 | 1626.8413 |
| **15** | GYSFVTTAER |  | 199-208 | 6.3 | 1130.56 | 1130.5482 |
| **16** | GYSFVTTAEREIVR |  | 199-212 | 5.5 | 1627.85 | 1627.8444 |
| **17** | GYSFVTTAEREIVRDIK |  | 199-215 | 5.3 | 1984.06 | 1984.0503 |
| **18** | GYSFVTTAEREIVRDIKEK |  | 199-217 | 6.4 | 2241.20 | 2241.1879 |
| **19** | EIVRDIKEK |  | 209-217 | 5.9 | 1129.66 | 1129.6581 |
| **20** | SYELPDGQVITIGNER |  | 241-256 | 5.7 | 1799.90 | 1790.8924 |
| **21** | SYELPDGQVITIGNERFR |  | 241-258 | 5.9 | 2094.07 | 2094.0620 |
| **22** | DLYANNVLSGGTTMYPGIADR | Phospho (ST) | 294-314 | 5.8 | 2308.05 | 2308.0321 |
| **23** | MQKEITALAPSTMKIK |  | 315-330 | 6.2 | 1790.00 | 1789.9920 |
| **24** | EITALAPSTMK |  | 318-328 | 5.7 | 1161.63 | 1161.6189 |
| **25** | IKIIAPPER |  | 329-337 | 5.8 | 1036.66 | 1036.6519 |
| **26** | IKIIAPPERK |  | 329-338 | 5.5 | 1164.75 | 1164.7468 |
| **27** | IIAPPERK |  | 331-338 | 5.7 | 923.57 | 923.5678 |
| **28** | QEYDEAGPSIVHRKCF |  | 362-377 | 5.6 | 1878.89 | 1878.8808 |

| **1** | M**CDDEEVTAL** | **VCDNGSGLVK** | **AGFAGDDAPR** | **AVFPSIVGRP** | **RHQGVMVGMG** |
| --- | --- | --- | --- | --- | --- |
| **51** | **QKDSYVGDEA** | **QSKRGILTLK** | **YPIEHGIITN** | **WDDMEKIWHH** | **TFYNELRVAP** |
| **101** | **EEHPTLLTEA** | **PLNPKANREK** | MTQIMFETFN | VPAMYVAIQA | VLSLYASGRT |
| **151** | TGIVLDAGDG | VTHNVPVYEG | YALPHAIMRL | DLAGR**DLTDY** | **LMKILTERGY** |
| **201** | **SFVTTAEREI** | **VRDIKEK**LCY | VALDFENEMA | TAASSSSLEK | **SYELPDGQVI** |
| **251** | **TIGNERFR**CP | ETLFQPSFIG | MESAGIHETA | YNSIMKCDID | IRK**DLYANNV** |
| **301** | **LSGGTTMYPG** | **IADRMQKEIT** | **ALAPSTMKIK** | **IIAPPERK**YS | VWIGGSILAS |
| **351** | LSTFQQMWIT | K**QEYDEAGPS** | **IVHRKCF** |  |  |

**Sequence coverage 58%**

**Table 10S**

|  | **Tropomyosin alpha-1 chain**  **TPM1_DANRE** | | | | | |
| --- | --- | --- | --- | --- | --- | --- |
|  | **Sequence** | **Modifications** | **Start-End** | **Δppm** | **Prec m/z** | **Theor m/z** |
| **1** | KKMQMLK |  | 6-12 | 5.8 | 906.53 | 906.5269 |
| **2** | KMQMLKLDKENALDR | 2 Oxidation (M) | 7-21 | 5.7 | 1864.97 | 1864.9624 |
| **3** | AEQAETDKKAAEERSK |  | 22-37 | 6.3 | 1790.90 | 1790.8884 |
| **4** | KAAEERSK |  | 30-37 | 5.5 | 918.51 | 918.5009 |
| **5** | SKQLEDDLVALQKK |  | 36-49 | 5.3 | 1614.92 | 1614.9066 |
| **6** | QLEDDLVALQKKLK |  | 38-51 | 6.4 | 1640.97 | 1640.9587 |
| **7** | ATEDELDK |  | 52-59 | 5.9 | 920.43 | 920.4213 |
| **8** | ATDAEGDVASLNRR |  | 78-91 | 5.7 | 1474.73 | 1474.7250 |
| **9** | LEEAEKAADESERGMK |  | 113-128 | 5.9 | 1792.85 | 1792.8387 |
| **10** | VIENRALK |  | 129-136 | 5.8 | 942.58 | 942.5736 |
| **11** | HIAEEADR |  | 153-160 | 6.2 | 940.45 | 940.4488 |
| **12** | EAETRAEFAERSVAK |  | 234-248 | 5.7 | 1693.86 | 1693.8509 |
| **13** | LEKTIDDLEDELYAQKLK |  | 249-266 | 5.8 | 2164.15 | 2164.1389 |
| **14** | TIDDLEDELYAQK |  | 252-264 | 5.5 | 1552.75 | 1552.7382 |

| **1** | MDAIK**KKMQM** | **LKLDKENALD** | **RAEQAETDKK** | **AAEERSKQLE** | **DDLVALQKKL** |
| --- | --- | --- | --- | --- | --- |
| **51** | **KATEDELDK**Y | SEALKDAQEK | LELAEKK**ATD** | **AEGDVASLNR** | **R**IQLVEEELD |
| **101** | RAQERLATAL | QK**LEEAEKAA** | **DESERGMKVI** | **ENRALK**DEEK | MELQEIQLKE |
| **151** | AK**HIAEEADR** | KYEEVARKLV | IVEGELERTE | ERAELNEGKC | SELEEELKTV |
| **201** | TNNMKSLEAQ | AEKYSAKEDK | YEEEIKVLTD | KLK**EAETRAE** | **FAERSVAKLE** |
| **251** | **KTIDDLEDEL** | **YAQKLK**YKAI | SEELDHALND | MTSI |  |

**Sequence coverage 48%**

**Table 11S**

|  | **Metalloendopeptidase OMA1**  **OMA1_DANRE** | | | | | |
| --- | --- | --- | --- | --- | --- | --- |
|  | **Sequence** | **Modifications** | **Start-End** | **Δppm** | **Prec m/z** | **Theor m/z** |
| **1** | THSAIRCCAQR |  | 22-32 | 6.0 | 1245.60 | 1245.5945 |
| **2** | TGAPAALRAPVVFQR |  | 86-100 | 6.3 | 1553.90 | 1553.8916 |
| **3** | APVVFQRTR |  | 94-102 | 5.9 | 1073.63 | 1073.6220 |
| **4** | GFHTSGRR |  | 103-110 | 5.8 | 917.48 | 917.4706 |
| **5** | RALPALPLLWMVLK |  | 111-124 | 5.9 | 1621.01 | 1621.0027 |
| **6** | PLQKIMAIILGRSIR |  | 124-139 | 5.7 | 1709.27 | 1709.2604 |
| **7** | KWWVALPANK |  | 139-149 | 5.9 | 1212.70 | 1212.6893 |
| **8** | KQLFREWSWRR |  | 150-160 | 5.6 | 1591.87 | 1591.8610 |
| **9** | EWSWRR |  | 155-160 | 5.7 | 919.46 | 919.4538 |
| **10** | TRLLVFSRK |  | 192-200 | 5.4 | 1119.71 | 1119.7002 |
| **11** | ELAQFNADAFMEEFK |  | 203-218 | 5.8 | 1789.82 | 1789.8107 |
| **12** | DSLIASSDPR |  | 219-228 | 5.2 | 1060.53 | 1060.5275 |
| **13** | DSLAALGHWIQGK |  | 334-346 | 5.9 | 1395.75 | 1395.7384 |
| **14** | LVQFLFDRPFSRK |  | 347-359 | 5.5 | 1652.94 | 1652.9276 |
| **15** | LEAEADQVGLQMAAK |  | 360-374 | 6.0 | 1573.80 | 1573.7895 |
| **16** | QLDRLIPEALELR |  | 417-429 | 5.5 | 1565.91 | 1565.9015 |
| **17** | LIPEALELR |  | 421-429 | 6.1 | 1053.64 | 1053.6308 |
| **18** | CNCPELPK |  | 432-439 | 6.2 | 903.41 | 903.4068 |
| **19** | CNCPELPKTDPR |  | 432-443 | 5.8 | 1372.64 | 1372.6353 |
| **20** | VVFNEAVR |  | 444-451 | 5.7 | 933.52 | 933.5158 |
| **21** | EQMLEKEEKNGK |  | 459-470 | 6.3 | 1462.73 | 1462.7211 |
| **22** | EEKNGKTQTGDMFP |  | 465-478 | 5.5 | 1581.73 | 1581.7219 |

| **1** | MQQTCIRLVK | LDMLSTLTRF | R**THSAIRCCA** | **QR**LFHCRPSL | FISARTYFIK |
| --- | --- | --- | --- | --- | --- |
| **51** | IDSSSLPKLK | GSVSFSASCV | SLGSSRLGLC | SSSFK**TGAPA** | **ALRAPVVFQR** |
| **101** | **TRGFHTSGRR** | **RALPALPLLW** | **MVLKPLQKIM** | **AIILGRSIRK** | **WWVALPANKK** |
| **151** | **QLFREWSWRR** | RWHFLGAGTG | LLFIASLFFF | THLDESPITG | R**TRLLVFSRK** |
| **201** | NFR**ELAQFNA** | **DAFMEEFKDS** | **LIASSDPR**HK | VVEQVVQILA | QRNQDIAEIS |
| **251** | AVPWTVHVVD | SPTMNAFVLP | NGEIFVFTGM | LNAVTDIHQL | TFILGHEMAH |
| **301** | ALIGHAAEQA | SLSHVVELLS | LVLLTAIWAV | CPR**DSLAALG** | **HWIQGKLVQF** |
| **351** | **LFDRPFSRKL** | **EAEADQVGLQ** | **MAAK**ACADVR | AGPVFWEQME | IFDQLSGQPT |
| **401** | MPEWLSTHPS | HQNRVR**QLDR** | **LIPEALELR**A | R**CNCPELPKT** | **DPRVVFNEAV** |
| **451** | **R**LVLEGKK**EQ** | **MLEKEEKNGK** | **TQTGDMFP** |  |  |

**Sequence coverage 45%**

**Table 12S**

|  | **Hypoxia-inducible factor 1-alpha**  **HIF1A_ONCMY** | | | | | |
| --- | --- | --- | --- | --- | --- | --- |
|  | **Sequence** | **Modifications** | **Start-End** | **Δppm** | **Prec m/z** | **Theor m/z** |
| **1** | VSSDRRKEK |  | 13-21 |  |  |  |
| **2** | EKSRDAAR |  | 20-27 |  |  |  |
| **3** | EKSRDAARCR |  | 20-29 |  |  |  |
| **4** | GKESEVFYELAQELPLPHSVTSNLDK |  | 31-56 |  |  |  |
| **5** | ASIMRLAISYLHMR |  | 57-70 |  |  |  |
| **6** | EMDSQLNGSYLK |  | 85-96 |  |  |  |
| **7** | AIEGFLMVLSEDGDMIYLSENVNK |  | 97-120 |  |  |  |
| **8** | SKEPNTER |  | 160-167 |  |  |  |
| **9** | MKCTLTNR | Oxidation (M) | 173-180 |  |  |  |
| **10** | MKCTLTNRGR |  | 173-182 |  |  |  |
| **11** | THHNLFAK |  | 292-299 |  |  |  |
| **12** | GQVSTGQYRMLAK |  | 300-312 |  |  |  |
| **13** | RGGFVWVETQATVIYNNK |  | 313-330 |  |  |  |
| **14** | MMLSLEQTEDMRPVK |  | 352-366 |  |  |  |
| **15** | MMLSLEQTEDMRPVK | 2 Oxidation (M) | 352-366 |  |  |  |
| **16** | KELEEEESSEPEVSPVLLK |  | 367-385 |  |  |  |
| **17** | SPELDVIKLFTR |  | 389-400 |  |  |  |
| **18** | DVMLPSTSDK |  | 454-463 |  |  |  |
| **19** | LDMVETLFAINPEPK |  | 527-541 |  |  |  |
| **20** | TVPQMDREISLR | Oxidation (M) | 645-656 |  |  |  |
| **21** | SLASQNAQR |  | 657-665 |  |  |  |
| **22** | SLASQNAQRKR |  | 357-667 |  |  |  |
| **23** | MSLSQAVGIGGLLQDHPGPGKK | Oxidation (M) | 669-690 |  |  |  |
| **24** | VSELSHADAPFNR |  | 693-705 |  |  |  |
| **25** | TILLLPTDLASR |  | 706-717 |  |  |  |
| **26** | LLGISSEGSGSPFTLPQLTR |  | 718-737 |  |  |  |
| **27** | YDCEVNAPVGGR |  | 738-749 |  |  |  |

|  |  |  |  |  |  |
| --- | --- | --- | --- | --- | --- |
| **1** | MDTGVVPEKK | SR**VSSDRRKE** | **KSRDAARCR**R | **GKESEVFYEL** | **AQELPLPHSV** |
| **51** | **TSNLDKASIM** | **RLAISYLHMR** | NLLSTDNEEE | QEER**EMDSQL** | **NGSYLKAIEG** |
| **101** | **FLMVLSEDGD** | **MIYLSENVNK** | CLGLAQIDLT | GLSVFEYTHP | CDHEELREML |
| **151** | VHRTGTSKK**S** | **KEPNTER**SFF | LR**MKCTLTNR** | **GR**TVNVKSAT | WKVLHCSDHV |
| **201** | RVHESPAEQI | PGGHKEPSVP | YLVLVCDPIP | HPSNIEAPLD | TKTFLSRHTL |
| **251** | DMKFTYCDER | ITELMGYDPE | DLLNRSVYEY | YHALDSDHLM | K**THHNLFAKG** |
| **301** | **QVSTGQYRML** | **AKRGGFVWVE** | **TQATVIYNNK** | NSQPQCVVCV | NYVLSGIEEE |
| **351** | K**MMLSLEQTE** | **DMRPVKKELE** | **EEESSEPEVS** | **PVLLK**EEK**SP** | **ELDVIKLFTR** |
| **401** | AVETQPLSSL | YDRLKEEPEA | LTLLAPAAGD | TIISLDFSSP | DSDILQKEVP |
| **451** | LYK**DVMLPST** | **SDK**LALPLSL | LPPSDQHLVP | NTSVDTTEVS | TGPDSSSTPG |
| **501** | SHSFTEPDSP | LDFCFPMESD | INAEFK**LDMV** | **ETLFAINPEP** | **K**TPFTLQAME |
| **551** | DLDLEMLAPY | IPMDDDFQLR | TLSPEEPLSC | GPAQPLECSS | LCSSVRLTQE |
| **601** | VHSYPGSPFN | APGSLTASPA | LAASPALAAP | EPADSPCPAS | LLTK**TVPQMD** |
| **651** | **REISLRSLAS** | **QNAQRKR**K**MS** | **LSQAVGIGGL** | **LQDHPGPGKK** | LK**VSELSHAD** |
| **701** | **APFNRTILLL** | **PTDLASRLLG** | **ISSEGSGSPF** | **TLPQLTRYDC** | **EVNAPVGGR**Q |
| **751** | LLLQGEELLS | ALDQVN |  |  |  |

**Sequence coverage 43%**

Table 13S

| Normoxia | | | | | |
| --- | --- | --- | --- | --- | --- |
|  | UniProtKB | Gene name (Danio rerio) | ZIFN ID | Protein | Enzyme Code |
| 1 | LDHA_DANRE | ldha | ZDB-GENE-991026-5 | L-lactate dehydrogenase A chain | [1.1.1.27](https://enzyme.expasy.org/EC/1.1.1.27) |
| 2 | G3P_DANRE | gapdh | ZDB-GENE-030115-1 | Glyceraldehyde-3-phosphate dehydrogenase  By similarity:GAPDH  By similarity: Peptidyl-cysteine S-nitrosylase GAPDH | 1.2.1.12  2.6.99.- |
| 3 | ALDCB_DANRE | aldocb | [ZDB-GENE-030821-1](http://zfin.org/cgi-bin/webdriver?MIval=aa-markerview.apg&OID=ZDB-GENE-030821-1) | Fructose-bisphosphate aldolase C-B | 4.1.2.13 |
| 5 | VTC1A_DANRE | atp6v1c1a | ZDB-GENE-030616-612 | V-type proton ATPase subunit C 1-A |  |
| 6 | Q6TH14_DANRE | eno3 | ZDB-GENE-031006-5 | Enolase 1, (Alpha) |  |
| 7 | OMA1_DANRE | oma1 | ZDB-GENE-091204-124 | Metalloendopeptidase OMA1, mitochondrial | 3.4.24.- |
| 8 | Q7ZU23_DANRE | actc1b | ZDB-GENE-000322-1 | Actin, alpha 1, skeletal muscle |  |
| 9 | TPM1_DANRE | tpma | ZDB-GENE-990415-269 | Tropomyosin alpha-1 chain |  |
| 10 | MYG_DANRE | mb | ZDB-GENE-040426-1430 | Myoglobin |  |
| 11 | HBB1_DANRE | ba1 | ZDB-GENE-990415-18 | Hemoglobin subunit beta-1 |  |

Table 14S

| Hypoxia | | | | | |
| --- | --- | --- | --- | --- | --- |
|  | UniProtKB | Gene name (Danio rerio) | ZIFN ID | Protein | Enzyme Code |
| 1 | LDHA_DANRE | ldha | ZDB-GENE-991026-5 | L-lactate dehydrogenase A chain | [1.1.1.27](https://enzyme.expasy.org/EC/1.1.1.27) |
| 2 | G3P_DANRE | gapdh | ZDB-GENE-030115-1 | Glyceraldehyde-3-phosphate dehydrogenase  By similarity:GAPDH  By similarity: Peptidyl-cysteine S-nitrosylase GAPDH | 1.2.1.12  2.6.99.- |
| 3 | ALDOB_DANRe | aldob | ZDB-GENE-030131-383 | Fructose-bisphosphate aldolase B | 4.1.2.13 |
| 4 | VTC1A_DANRE | atp6v1c1a | ZDB-GENE-030616-612 | V-type proton ATPase subunit C 1-A |  |
| 5 | Q6TH14_DANRE | eno3 | ZDB-GENE-031006-5 | Enolase 1, (Alpha) |  |
| 6 | OMA1_DANRE | oma1 | ZDB-GENE-091204-124 | Metalloendopeptidase OMA1, mitochondrial | 3.4.24.- |
| 7 | Q7ZU23_DANRE | actc1b | ZDB-GENE-000322-1 | Actin, alpha 1, skeletal muscle |  |
| 8 | TPM1_DANRE | tpma | ZDB-GENE-990415-269 | Tropomyosin alpha-1 chain |  |
| 9 | MYG_DANRE | mb | ZDB-GENE-040426-1430 | Myoglobin |  |
| 10 | HBB1_DANRE | ba1 | ZDB-GENE-990415-18 | Hemoglobin subunit beta-1 |  |
| 11 | HIF1N_DANRE | hif1an | ZDB-GENE-030826-19 | Hypoxia-inducible factor 1-alpha inhibitor | 1.14.11.n4 |

**Alignment 1**

ALDCB (Fructose-bisphosphate aldolase C-B, “gene name aldoc”) AND ALDOB (Fructose-bisphosphate aldolase B, “gene name aldob”) Danio Rerio

(*) indicates positions which have a single, fully conserved residue

(:) indicates conservation between groups of strongly similar properties

(.) indicates conservation between groups of weakly similar properties

In yellow are highlighted the region with no similar properties

Identical Positions: 269

Identity: 73.901%

Similar positions: 63

SP|Q8JH70|ALDCB_DANRE MTHQYPALTAEQKKELQDIAQRIVAPGKGILAADESTGSMAKRLNPIGVENTEENRRLYR 60

SP|Q8JH71|ALDOB_DANRE MTHQFPALSTEQKKELATIAERIVAPGKGILAADESTGTMAKRFQKINVENTEENRRSFR 60

****:***::****** **:*****************:****:: *.********* :*

SP|Q8JH70|ALDCB_DANRE QLLFSADERIDKCIGGVIFFHETLYQNTDDGTNFAQLIKDRGIVVGIKVDKGVVPLAGTN 120

SP|Q8JH71|ALDOB_DANRE DLLFSVDDSISESIGGVILFHETLYQKSDKGVLFPKVIKDKGIVVGIKVDKGTAGLAGTD 120

:****.*: *.:.*****:*******::*.*. * ::***:***********.. ****:

SP|Q8JH70|ALDCB_DANRE GETTTQGLDGLSERCAQYKKDGADFAKWRSVLKISDTTPSELAIMENANVLARYASICQQ 180

SP|Q8JH71|ALDOB_DANRE GETTTQGLDGLSERCAQYKKDGCDFAKWRCVLKISDSCPSALGIAENANVLARYASICQQ 180

**********************.******.******: ** *.* ***************

SP|Q8JH70|ALDCB_DANRE NGIVPIVEPEILPDGEHDLKRCQYVTEKVLAACYKALSDHHVYLEGTLLKPNMVTAGHSC 240

SP|Q8JH71|ALDOB_DANRE NGLVPIVEPEILPDGDHDLKQCQYATEKVLAAVYKALSDHHVYLEGTLLKPNMVTAGHSC 240

**:************:****:***.******* ***************************

SP|Q8JH70|ALDCB_DANRE PTKYSSEEIAMATVTALRRTVPPAVSGVTFLSGGQSEEEASVNLNSINNCPLAKPWPLTF 300

SP|Q8JH71|ALDOB_DANRE TKKYTPLEVAMATVTALRRTVPAAVPGICFLSGGQSEEEASLNLNAMNQLSLHRPWKLSF 300

.**: *:************* ** *: ************:***::*: * :** *:*

SP|Q8JH70|ALDCB_DANRE SYGRALQASALSAWRGAKSNEKAATEEFIKRAEANGLAAQGKYVSSGTCGAAG-QSLYVA 359

SP|Q8JH71|ALDOB_DANRE SYGRALQASALSAWKGQAANKKASQDAFVTRAKINSLASKGEYKPSGQAGQASTQSLFTA 360

**************:* :*:**: : *:.**: *.**::*:* ** .* *. ***:.*

SP|Q8JH70|ALDCB_DANRE NHAY 363

SP|Q8JH71|ALDOB_DANRE SYTY 364

.::*

**Alignment 2**

ALDC (Fructose-bisphosphate aldolase C, “gene name aldoc” Carassius Auratus) and ALDOB (Fructose-bisphosphate aldolase B, “gene name aldob” Danio Rerio)

Identical Positions: 269

Identity: 73.901%

Similar positions: 63

SP|Q8JH71|ALDOB_DANRE MTHQFPALSTEQKKELATIAERIVAPGKGILAADESTGTMAKRFQKINVENTEENRRSFR 60

SP|P53448|ALDOC_CARAU MTHQYPALTTEQKRELQDIAQRIVAPGKGILAADESTGSMAKRLNPIGVENTEENRRLYR 60

****:***:****:** **:*****************:****:: *.********* :*

SP|Q8JH71|ALDOB_DANRE DLLFSVDDSISESIGGVILFHETLYQKSDKGVLFPKVIKDKGIVVGIKVDKGTAGLAGTD 120

SP|P53448|ALDOC_CARAU QLLFTADERMDKCIGGVIFFHETLYQKADDGTPFAKMIKDRGIVVGIKVDKGVVPLAGTN 120

:***:.*: :.:.*****:********:*.*. * *:***:***********.. ****:

SP|Q8JH71|ALDOB_DANRE GETTTQGLDGLSERCAQYKKDGCDFAKWRCVLKISDSCPSALGIAENANVLARYASICQQ 180

SP|P53448|ALDOC_CARAU GETTTQGLDGLSERCAQYKKDGADFAKWRSVLKISETSPSELAIMENANVLARYASICQQ 180

**********************.******.*****::.** *.* ***************

SP|Q8JH71|ALDOB_DANRE NGLVPIVEPEILPDGDHDLKQCQYATEKVLAAVYKALSDHHVYLEGTLLKPNMVTAGHSC 240

SP|P53448|ALDOC_CARAU NGIVPIVEPEILPDGDHDLKRCQYVTEKVLAACYKALSDHHVYLEGTLLKPNMVTAGHSC 240

**:*****************:***.******* ***************************

SP|Q8JH71|ALDOB_DANRE TKKYTPLEVAMATVTALRRTVPAAVPGICFLSGGQSEEEASLNLNAMNQLSLHRPWKLSF 300

SP|P53448|ALDOC_CARAU PTKFSNQEIAMATVTALRRTVPPAVTGVTFLSGGQSEEEASINLNAINNCPLTKPWALTF 300

.*:: *:************* ** *: ************:****:*: * :** *:*

SP|Q8JH71|ALDOB_DANRE SYGRALQASALSAWKGQAANKKASQDAFVTRAKINSLASKGEYKPSGQAGQASTQSLFTA 360

SP|P53448|ALDOC_CARAU SYGRALQASALSAWRGVKENEKAATEEFLKRAEANGLAAQGKYVSSGMDGSAG-QSLYVA 359

**************:* *:**: : *:.**: *.**::*:* ** *.*. ***:.*

SP|Q8JH71|ALDOB_DANRE SYTY 364

SP|P53448|ALDOC_CARAU NHAY 363

.::*
